# The fall of the summer truffle: Recurring hot, dry summers result in declining fruitbody production of *Tuber aestivum* in Central Europe

**DOI:** 10.1111/gcb.16424

**Published:** 2022-10-06

**Authors:** Brian S. Steidinger, Ulf Büntgen, Uli Stobbe, Willy Tegel, Ludger Sproll, Matthias Haeni, Barbara Moser, István Bagi, José‐Antonio Bonet, Marc Buée, Benjamin Dauphin, Fernando Martínez‐Peña, Virginie Molinier, Roman Zweifel, Simon Egli, Martina Peter

**Affiliations:** ^1^ Department of Ecology University of Konstanz Konstanz Germany; ^2^ Swiss Federal Institute for Forest, Snow and Landscape Research WSL Birmensdorf Switzerland; ^3^ Global Change Research Centre (Czech Globe) Brno Czech Republic; ^4^ Department of Geography University of Cambridge Cambridge UK; ^5^ Department of Geography, Faculty of Science Masaryk University Brno Czech Republic; ^6^ Deutsche Trüffelbäume Bodman Germany; ^7^ Forest Growth Albert‐Ludwigs University Freiburg Germany; ^8^ NEFAG Zrt. Szolnok Hungary; ^9^ Forest Science and Technology Centre of Catalonia Solsona Spain; ^10^ Laboratory of Excellence ARBRE, INRAE‐Grand Est, Interactions Arbres/Microorganismes INRAE, UMR 1136 INRAE‐University of Lorraine Champenoux France; ^11^ Agrifood Research and Technology Centre of Aragon CITA Zaragoza Spain; ^12^ European Mycological Institute EGTC‐EMI Soria Spain

**Keywords:** climate change, drought extremes, ecological niche, global warming, mycorrhizal fungi, truffles

## Abstract

Global warming is pushing populations outside their range of physiological tolerance. According to the environmental envelope framework, the most vulnerable populations occur near the climatic edge of their species' distributions. In contrast, populations from the climatic center of the species range should be relatively buffered against climate warming. We tested this latter prediction using a combination of linear mixed effects and machine learning algorithms on an extensive, citizen‐scientist generated dataset on the fruitbody productivity of the Burgundy (aka summer) truffle (*Tuber aestivum* Vittad*.*), a keystone, ectomycorrhizal tree‐symbiont occurring on a wide range of temperate climates. *T. aestivum*'s fruitbody productivity was monitored at 3‐week resolution over up to 8 continuous years at 20 sites distributed in the climatic center of its European distribution in southwest Germany and Switzerland. We found that *T. aestivum* fruitbody production is more sensitive to summer drought than would be expected from the breadth of its species' climatic niche. The monitored populations occurring nearly 5°C colder than the edge of their species' climatic distribution. However, interannual fruitbody productivity (truffle mass year^−1^) fell by a median loss of 22% for every 1°C increase in summer temperature over a site's 30‐year mean. Among the most productive monitored populations, the temperature sensitivity was even higher, with single summer temperature anomalies of 3°C sufficient to stop fruitbody production altogether. Interannual truffle productivity was also related to the phenology of host trees, with ~22 g less truffle mass for each 1‐day reduction in the length of the tree growing season. Increasing summer drought extremes are therefore likely to reduce fruiting among summer truffle populations throughout Central Europe. Our results suggest that European *T. aestivum* may be a mosaic of vulnerable populations, sensitive to climate‐driven declines at lower thresholds than implied by its species distribution model.

## Graphical abstract

We worked with citizen scientists to monitor the productivity of the widely distributed summer truffle (*Tuber aestivum*) over eight years. We found that hot, dry summers substantially reduce truffle yields. Populations from the center of the species' range cannot tolerate conditions experienced by populations at the hotter extremes, suggesting local adaptation. This may exacerbate summer truffles response to climate change, sensitizing populations to the increasing the frequency of summer drought.

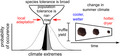

## 1 INTRODUCTION

Global warming is pushing populations outside their range of physiological tolerance, leading to concerted poleward migration and elevational climbing among a broad array of plants, animals, and fungi attempting to track their thermal niches (Chen et al., 2011; Diez et al., 2020; Lenoir & Svenning, 2015). The resulting local species extirpation and migration can disrupt interaction networks, resulting in loss of ecosystem resiliency and associated services (Ebenman & Jonsson, 2005). Accurately predicting these shifts is thus essential for preparing environmental stakeholders to cope with the altered composition of their ecological communities—particularly when keystone species are displaced.

True truffles are fungi belonging to the genus *Tuber*, whose species form symbiotic associations with roots of a wide range of ectomycorrhizal tree species (Benucci et al., 2011; Stobbe et al., 2012). Truffle species include the most valuable fungi on earth due to their use in haute cuisine (Reyna & Garcia‐Barreda, 2014). As ectomycorrhizal fungi, truffles grant their hosts access to water and nutrients, protect roots from pest and disease, and interact with and sometimes kill understory, non‐host plants (Gryndler et al., 2014; Streiblová et al., 2012; Taschen et al., 2020). Much remains unknown about the life history of these iconic species due to a lack of available data from natural truffle populations. However, available data on black truffle production in Spain, Italy, and France suggest that truffle species may be vulnerable to global climate change (Baragatti et al., 2019; Büntgen et al., 2011, 2012).

The black Périgord truffle (*T. melanosporum* Vittad.) is known to be on the decline in its natural range. During the golden age of Périgord truffle production in the 19th century, France produced up to 1588 tons year^−1^ of commercial *T. melanosporum*. Today, the same regions produce 10–50 tons year^−1^ (productivity data from Baragatti et al. (2019), Reyna‐Domenech and García‐Barreda (2009)). Although wars and economy account for a substantial part of these declines, they do not tell the whole story. Declines in Mediterranean harvests in France and Italy from 1970 to 2006 are best predicted by a trend of hotter, drier summers (Büntgen et al., 2011, 2012). Existing Périgord truffle habitat is becoming unsuitable, consistent with the climate envelope migrating northwards from the Mediterranean area to Central Europe (Čejka et al., 2020).

Similar data on fruitbody production are unavailable for the congeneric Summer truffle (*Tuber aestivum* Vittad. Syn. *Tuber uncinatum* Chatin). Summer truffle populations are substantially more widely distributed than Mediterranean Périgord truffles, producing fruitbodies in tree stands ranging from the hot, dry climates of Spain to the cold, temperate climates of Scandinavia (Molinier, Peter, et al., 2016; Stobbe et al., 2012, 2013). Together with its broad ecological niche, the Summer truffle's long fruitbody production period and high market value make it attractive for cultivation and wild harvesting (Stobbe et al., 2013).

The ecological niche is the core principle describing how species respond environmental change (Grinnell, 1914). Accordingly, species occur within a restricted range of tolerable environmental values (Hutchinson, 1959). In practice, these tolerable conditions are approximated using ecological niche and/or species distribution models (Pearson & Dawson, 2003). In these models, species occurrence data are used to describe its environmental envelope (a niche by another name). The models are then used to predict occurrence probability across space (Elith et al., 2011; Soberón & Nakamura, 2009) and forward in time, forecasting species' responses to climate change (Beaumont et al., 2005; Dyderski et al., 2018; Harrison et al., 2006; Kane et al., 2017; Thomas et al., 2004).

**FIGURE 1:**
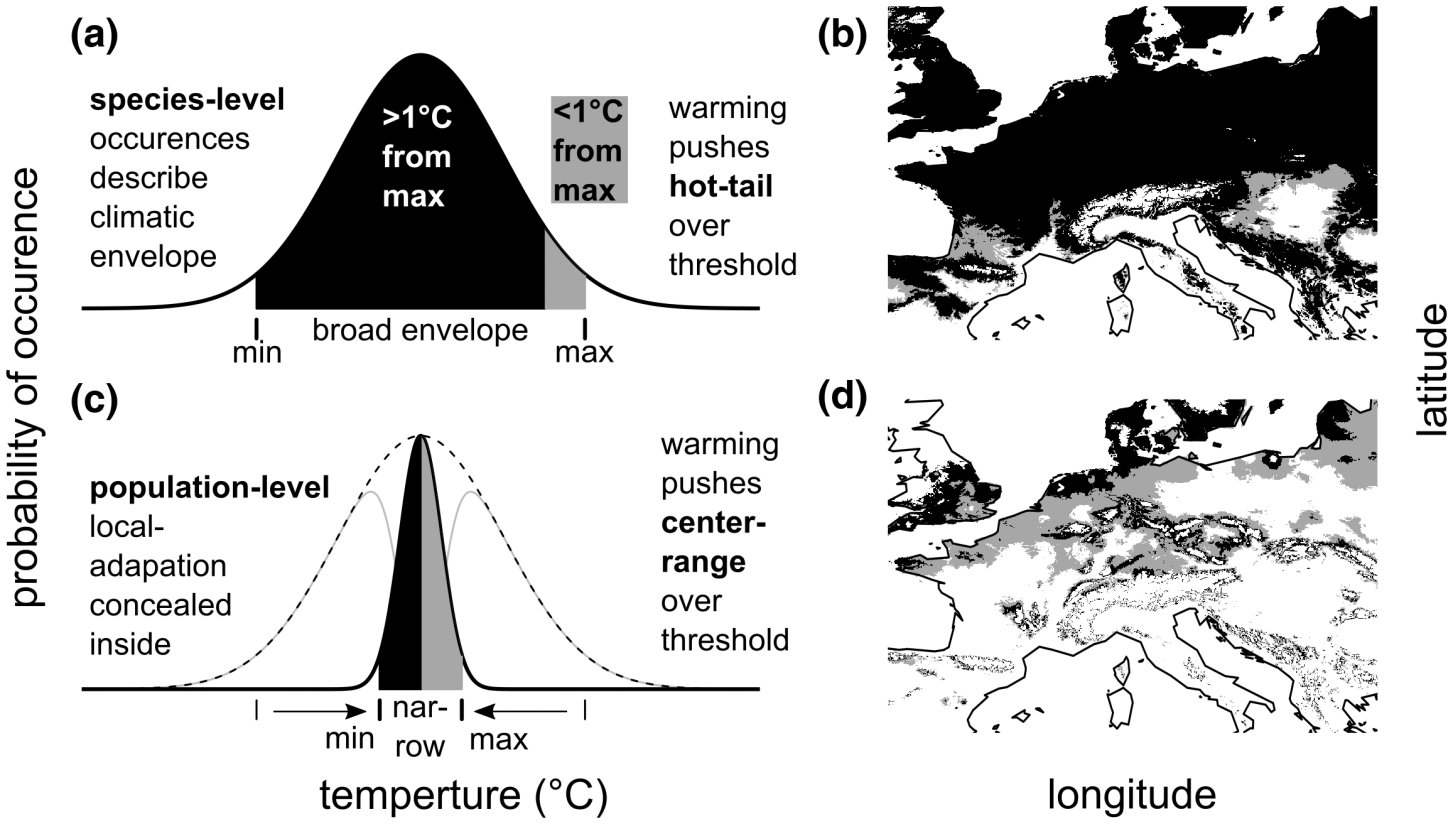
Conceptual figure, demonstration how local adaptation can sensitize populations to climate change. (a) Species‐level occurrences describe a climate envelope. Individuals <1°C from the max temperature are vulnerable to warming (gray). (b) These at risk‐individuals are limited to the hottest, southern range limits (gray). By contrast, if the species climate‐envelope conceals (c) locally adapted populations, then (d) at‐risk individuals occur in the center of the species' geographic and climatic range. The climate envelope is drawn from temperature of warmest quarter distributions, with species and population mean of 16°C and envelope extending ±2 standard deviations (2.0°C and 0.5°C for species and populations, respectively).

Over a sufficiently broad gradient of temperature, theory predicts and observation corroborates that climatic envelopes are unimodal (Austin, 2007; Chen et al., 2011; Steinbauer et al., 2018; Figure 1a). Thus, warming pushes populations from their species' hottest suitable habitats beyond their climatic optimum (Pucko et al., 2011; Reich et al., 2015) (Figure 1b). By contrast, populations sheltered near the center of their climate distributions are relatively stable, as they require larger temperature increases to push them beyond the species' tolerable limits (Araújo et al., 2005; Thomas et al., 2004). Accurately placing individuals within their climatic envelope is, therefore, essential to predicting their response to climate warming.

Pre‐empting the use of species distribution modeling techniques is the decision to group occurrence data by species. Such species‐level occurrence bagging assumes that each individual can tolerate climate conditions experienced across the species' entire geographic range (Figure 1a,b). By contrast, local adaptation can sensitize populations to conditions that occur within a species' geographic range (Figure 1c,d [Dongmo et al., 2021; Kaspari et al., 2015; Nadeau et al., 2017; Somero, 2010]). Thus, even center‐of‐range populations have been found to decline in fitness due to weather events, like summer drought, that are unusual by local climate standards yet still fall within the long‐term average conditions experienced by the species across its entire distribution (Atkins & Travis, 2010; Kelly et al., 2012; Knutzen et al., 2017). It is as though what appear to be single, resilient species are really mosaics of vulnerable populations.

Further exacerbating this local vulnerability is the increase in the frequency of extreme weather. Throughout continental Europe, climate change is increasing the frequency extreme hot summers (Alexander, 2011; Christidis et al., 2015). Accordingly, summer temperatures historically experienced once a century are now expected to occur twice a decade. Likewise, anomalies like the 2003 European heat wave, which historically occurred once every 1000 years, are now predicted to occur twice a century (Christidis et al., 2015). This begs the question of how safe center‐of‐range populations will react to increasing summer drought when sensitized (i.e., locally adapted); whether threats to the resilience of keystone species may be overlooked because those species appear to fill a broad climatic niche.

In the absence of local adaptation, summer truffles should be insensitive to climate warming relative to Périgord truffles, at least among populations at the center of the species' geographic range (Figure 1a,b). Alternatively, locally adapted could sensitize summer truffle populations to the recent trend of hot, dry summer than a single species distribution model would predict (Figure 1c,d). Only continuous monitoring data of fruitbody production in natural truffle populations, hereto lacking, can distinguish between these possibilities. Moreover, observation at a single time point may result in poor temporal extrapolation. Therefore, it is critical to generate time series of data suitable for generating model extrapolation across both space in time.

Here we present 8 years (2011–2018) of citizen science monitoring data on the fruitbody production of naturally occurring *T. aestivum* populations. Using machine learning, we modeled how truffle productivity is influenced by soil physicochemical, fungal meta‐community, climate, and host tree phenology. Additionally, we characterized the broad species climatic envelope of European summer truffles. By comparing the sensitivity of summer truffle fruitbody productivity to interannual climate trends, we test whether these center‐of‐range, keystone populations are locally sensitized to climate change.

## 2 MATERIALS AND METHODS

### 2.1 Site selection and truffle monitoring

**FIGURE 2:**
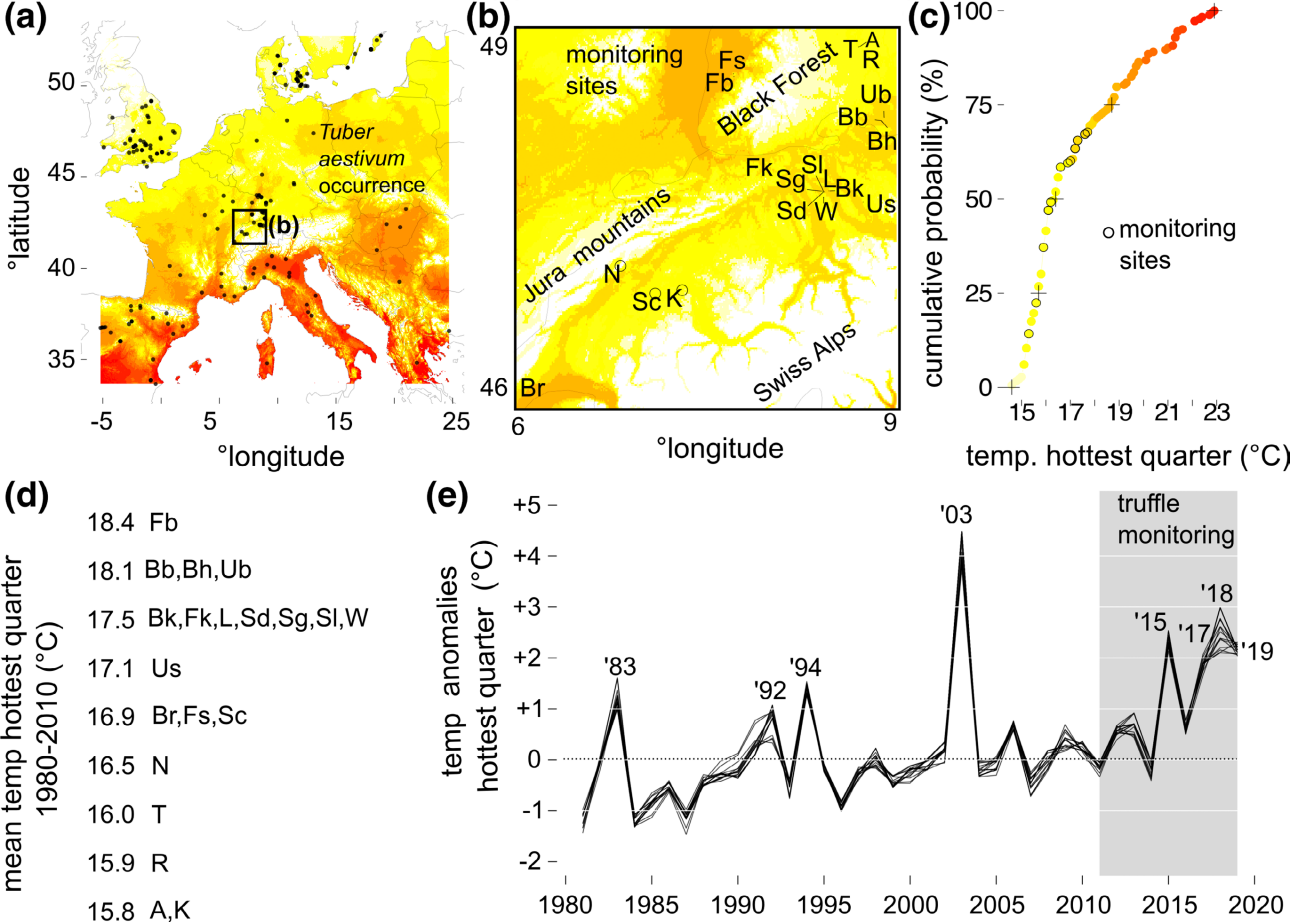
Truffle monitoring sites from this study come from the geographic and center of the species' European range, potentially buffering them from a sequence of summer hot temperature anomalies. (a) Species occurrences in Europe, with an inset (b) giving the location of the monitoring sites from this study and (c) cumulative distribution of the 30‐year mean temperature of the warmest quarter for all occurrences, inclusive of monitoring sites (circles with black borders) (heat‐scale colors are the same in (a), (b) and (c), + symbols indicate 0, 25, 50, 75, and 100 quantiles). (d) The mean temperature of the hottest quarter (1980–2010) for all monitoring sites from (b) listed in tabular form. (e) Time series of temperature anomalies for the hottest quarter of the year, with the 8‐year monitoring period shaded in gray. Map lines delineate study areas and do not necessarily depict accepted national boundaries.

We selected a total of 20 natural *T. aestivum* populations from within the center of its known natural geographic distribution to monitor truffle fruitbody production (Figure 2a). These populations were defined as spatially restricted locations of known truffle occurrences ranging in size from 20 to 1000 m^2^, depending on the contiguous area under compatible host trees where trained truffle dogs found *T. aestivum* fruitbodies.

Monitoring occurred every 3 weeks throughout the whole year by citizen scientists using dogs trained to scent summer truffles (see Table 1 for the years where monitoring data are available for each site). At each site, only one and the same citizen‐dog team performed the monitoring throughout the whole project. For all belowground fruitbodies detected by the truffle dogs, the soil was roughly removed and the species identity, mass (g), and degree of maturity were assessed. The degrees of maturity were divided into the following qualitative categories:

1. unripe: white, hard, no or indistinct odor
2. semi ripe: light beige‐light brown, hard, indistinct (nutty) odor
3. ripe: brown to dark brown, hard, intense odor

**TABLE 1:** Summary of site characteristics for 20 sites used in the analysis in descending order of the max truffle mass year^−1^

Site	Location (°E, °N)	Plot size (m^2^)	Dominant tree species	Total truffle mass (g year^−1^)			Monitoring years (20XX, truff / dendro)	pH (H_2_O)	Bare soil (%)
				Min	Mean	Max			
Fs	7.80, 47.94	150	*Fagus sylvatica*	1611.7	3089.2	6757.8	11,**12,13,14,**15,**16,** **12,13,14,** **16,**17**,**18	7.5	75.0
Ub	8.90, 47.75	800	*F. sylvatica*	0.0	1402.3	4158.3	12,13,14,15,16,17,18	7.7	0.1
Bb	8.88, 47.73	1000	*F. sylvatica*	971.0	2647.5	3909.8	11,12,13,14,15,16,17,18	7.4	0.0
Sl	8.46, 47.39	25	*Carpinus betulus*	38.0	1629.6	3233.0	12,13,14,15,16,17,18	8.2	40.0
R	8.79, 48.03	900	*F. sylvatica*	851.9	1998.1	3130.3	11,**12,13,14** **12,13,14**,15	7.8	2.0
Br	6.28, 46.46	300	*C. betulus*	58.0	812.1	2501.0	**13,14**,15,16,17,18 **13,14**	6.5	20.0
K	7.36, 46.88	400	*F. sylvatica*	336.9	1125.2	2388.8	**13,14,15,16,17,18** **13,14,15,16,17,18**	6.7	2.0
T	8.65, 48.07	400	*Corylus avellana*	1538.4	1613.6	1688.8	11,12	7.2	NA
Sd	8.46, 47.39	100	*F. sylvatica*	0.0	626.7	1658.0	12,13,14,15,16,17,18	8.0	2.0
Bk	8.49, 47.40	100	*Ostrya carpinifolia*	430.0	879.1	1591.0	16,17,18	NA	NA
L	8.50, 47.39	400	*C. betulus*	957.0	1216.0	1473.6	16,17,18	NA	NA
Bh	8.89, 47.73	100	*C. avellana*	218.0	716.7	1242.0	11,**12**,13,14,15 **12**	7.7	NA
W	8.46, 47.36	100	*F. sylvatica*	224.0	726.2	1141.1	16,17,18	NA	NA
A	8.71, 48.09	100	*Picea abies*	568.8	774.3	1030.0	11,**12,13,14** **12,13,14**,15,16	6.1	0.0
Fk	7.96, 47.50	400	*F. sylvatica*	0.0	331.1	752.9	**13,14,15,16,17,18** **13,14,15,16,17,18**	8.1	2.0
Us	8.75, 47.34	300	*F. sylvatica*	656.2	656.2	656.2	**13** **13**,14,15, 17	4.8	0.0
Fb	7.79, 47.96	150	*C. betulus*	8.2	297.0	567.5	11,12,13,14,15	8.1	30.0
Sc	7.16, 46.87	400	*F. sylvatica*	43.2	193.3	381.4	**13,14,15,16,17,18** **13,14,15,16,17,18**	6.4	1.0
N	6.91, 47.00	400	*F. sylvatica*	0.0	120.9	227.0	**13,14,15,16,17,18** **13,14,15,16,17,18**	6.2	1.0
Sg	8.45, 47.39	400	*F. sylvatica*	0.0	49.9	158.0	11,12,**13,14,15,16,17,18** **13,14,15,16,17,18**	6.0	5.0

*Note*: For monitoring years, black numbers refer to years where truffle monitoring took place, green numbers to years where dendrology data (e.g., water deficit) measurements were available for at least one tree. Bolded years emphasize overlap within a site of truffle and dendrology data.

### 2.2 Spatial–temporal climate distributions of *T. aestivum*

We defined a climate envelope for European *T. aestivum* using georeferenced occurrence records from the Global Biodiversity Information Facility (GBIF.org) supplemented with 33 geospatial *T. uncinatum* records from Wedén et al. (2005), and our own 20 monitoring sites (Figure 2a).

All statistical analyses were conducted in R Studio (Version 2022.2.0.443) (RStudio Team, 2022). GBIF occurrences were checked against common spatial mis‐placement using the R package “CoordinateCleaner” (Zizka et al., 2019).

### 2.3 Climate means and time series

We tracked the historical annual climate for each monitoring site by extracting monthly mean temperature at 2 m for the years 1980–2018 from the ERA‐5 reanalysis dataset (Hersbach et al., 2020). For each year, we calculated the mean temperature for the months June, July, and August. We then compared these temporal climate records to the spatial mean climate values experienced by European *T. aestivum*. We extracted 30‐year mean temperature of the hottest quarter for all *T. aestivum* occurrence coordinates from WorldClim 2.0 (inclusive of the monitoring sites, rasters at 1 km resolution; Fick & Hijmans, 2017).

**FIGURE 3:**
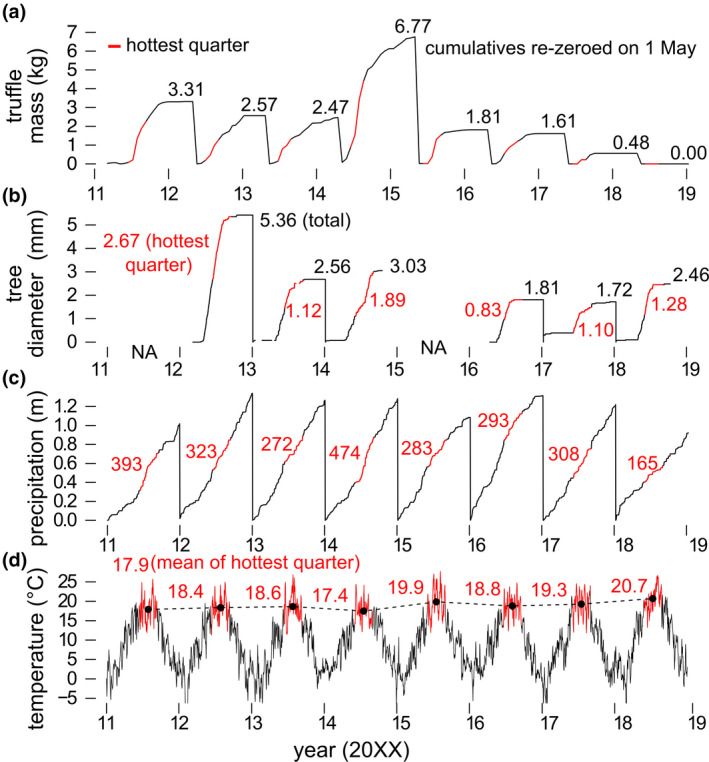
Comparison of inter‐annual climate and host‐tree growth may account for inter‐annual variability in truffle mass. Time series of (a) truffle productivity (cumulative), (b) tree growth (cumulative max value), (c) precipitation (cumulative), and (d) temperature for one site, Fs.

To track time series of air temperature at 2 m and total precipitation during the monitoring period, we downloaded ERA‐5 re‐analysis hourly weather data for the period of 2011–2018, covering the extent of the map inset in Figure 2a (5–10°E, 45–50°N) (Copernicus Climate Change Service, 2019, p. 5). We extracted data for each of the sites in the analysis (Table 1) from rasters at roughly 9 km resolution (0.1 decimal degrees). Hourly data were averaged first to daily values. The temperature of the warmest quarter was calculated by finding the hottest temperature averaged over 90 consecutive days each year (Figures 3d and 5). Similarly, for precipitation sum of the warmest quarter, the sum of precipitation was calculated over the same period (Figures 3c and 6e).

### 2.4 Site characteristics

Site characteristics refer to the climatic, vegetational, soil physicochemical, and soil fungal meta‐community variables that have one value per site (with no interannual variability). Besides climate variables derived from interpolated global rasters, the remaining variables were collected in situ at the monitoring sites (Supplementary Information).

### 2.5 Dendrology data

For a subset of sites, point dendrometers (DR20, Ecomatik) provided hourly stem diameter measurements (e.g., Figure 3b) for up to four potential *T. aestivum* host trees. Data for at least one tree were available for 52 site × year combinations, representing 11 sites. Eight truffle monitoring sites had ≥ three overlapping annual truffle mass and dendrology measurements (for a total of 39 measurements, see Table 1 for site × year combinations with available data). Thus, these eight sites were the only ones with sufficient data to compare dendrology and truffle data.

### 2.6 Total truffle mass, truffle yield, and variance partitioning

Annual truffle mass was calculated by summing the mass of all truffles from May 1st to April 30th of the following year. The April 30th cut‐off was chosen as it is the yearly low point of truffle productivity across sites. Because sites differed in size (Table 1), we used two different metrics of truffle productivity depending on whether we sought to explain variability within a site only (linear mixed effects models) or both within and among sites (random forest models). To account for potential artifacts due to differences in among sites in the sampling effort, we included the number (#) of surveys per truffle year as a covariate in our analyses. Only sites × year combinations with ≥12 surveys were included in our analyses. For the within‐site analyses, we used annual truffle mass in g year^−1^ and accounted for differences in site size by fitting random slopes and intercepts for each site. For the within‐ and among‐site random forest analyses, we divided annual truffle mass by the size of the plot to give truffle yields in g m^−2^ year^−1^.

We decomposed variance in truffle yield (g m^−2^ year^−1^) into the sum of covariance of log‐transformed yields and truffle numbers and mean truffle mass, respectively (Supplementary Information).

### 2.7 Host tree growing season and fungal fruiting season

To calculate the lengths of the growing and fruiting seasons for host trees and truffles, S‐shaped Gompertz functions were fit to the relationship between the numerical day of the year vs tree diameter and cumulative fruitbody production, respectively (Čufar et al., 2008). The fit parameters were used to calculate the start and end dates of tree growth and truffle fruiting as well as the day of the year associated with peak rate of increase in tree diameter and truffle fruit mass (Supplementary Information).

### 2.8 Tree growth and tree water deficit

Trees experience a regular cycle of expansion (at night, when water is taken up) and contraction (in the daytime, when water is lost to transpiration) (Zweifel et al., 2005, 2016). When sufficient water is available (and other conditions promote growth), tree expansion exceeds contraction; when dry conditions constrain tree growth, contraction exceeds expansion. This daily excess of contraction is termed tree water deficit (Δ*W*). It is measured with a point dendrometer using the following equation:

(1) $$\Delta W=W_{\max}-W_{\mathrm{act}},$$

where *W*_act_ is the actual diameter and *W*_max_ is calculated as the maximum diameter from *t* = 0 (12:00 am on January 1) to the present. By definition, *W*_max_ is the largest diameter over a given period of observation, such that *W*_max_ ≥ *W*_act_ and Δ*W* ≤ 0. To distinguish between water deficits that occur only during the daytime (and are relieved before the following dawn), we calculated seasonal averages of the daily maximum values of ΔW. The total summer water deficit was calculated as the mean water deficit value over all the trees for the 3‐month period from June to August.

### 2.9 Linear mixed‐effects models

We analyzed causes to the interannual decline in annual truffle mass (g year^−1^) within each site using linear mixed‐effects models and backwards model selection (“lme4” package) (Bates et al., 2015, p. 4). First, we built a model with both temperature of the hottest quarter and precipitation sum of the hottest quarter as fixed effects. Next, we stripped the model of terms with non‐significant *p*‐values (*p* > .05), including the covariate describing the number of samples per year. We performed a similar, but separate, analysis for the fixed effects of tree water‐deficit and tree growth on annual truffle mass (g year^−1^), as there were fewer site × year combinations with available data (Table 1). As before, random slopes and intercepts were fit for each site and statistical significance was assessed at *p* = .05. Total *R* ^2^ values were decomposed into total proportion of variance explained by the model (conditional *R* ^2^_conditional_) and the proportion of variance explained by the fixed effects alone (marginal *R* ^2^_marginal_).

### 2.10 Random forest model selection and validation

The machine learning algorithm random forest was used in R (package “randomForest”; Liaw & Wiener, 2002) to predict variability in productivity among sites and among years. Relative to other multiple regression models, random forests have been shown to be less prone to over‐fitting, more robust to co‐linearity among predictors (Matsuki et al., 2016) and more capable of identifying complex interactions among predictor variables (Strobl et al., 2009).

Model selection was performed as follows: first, we included all soil physicochemical, meta‐community, dendrological and climatic variables in our models; second, variables were sorted according to importance metrics (inc node purity and % mean squared error); third, we compared model performance (% variability explained) as predictors were removed from the model, starting with the least important. Our final model included only those variables beyond the inflection point of diminishing returns on performance (Figure 6a; Table S1). To visualize the effects of individual variables on annual truffle productivity, we extracted the partial feature contributions using the “forestFloor” package in R (Welling et al., 2016).

We cross validated our models using k‐fold cross‐fold evaluation. Data were separated into *k* = 10 partitions and model performance was gauged by summarizing the coefficient of determination for 1000 random folds.

## 3 RESULTS

### 3.1 Spatial–temporal climate distributions of European *T. aestivum*

The combined *T. aestivum* and *T. uncinatum* occurrences show a distribution across Europe, from Southern Spain to Scandinavia and northern England (Figure 2a). The 20 truffle monitoring sites are near the center of the pan‐European distribution, occurring within the borders of Switzerland and southwest Germany (Baden‐Wuerttemberg, Figure 2b).

The 30‐year (1980–2010) mean and median temperature of the hottest quarter of all European *T. aestivum* occurrences were 17.27 and 16.40°C, respectively. The truffle monitoring sites have average temperatures during the hottest quarter that fall in the lower 75% of the European *T. aestivum* records (18.50°C, Figure 2d), with the three coldest sites having average temperatures of the hottest quarter less than the bottom 25% (15.80°C, sites A, K, and R in Figure 2d). Thus, the range of temperatures separating the truffle monitoring sites from the climatic edge of the European distribution—which indicates the range of temperature anomalies necessary to put an individual site outside the mean climate distribution of the species—ranges from 4.4 to 6.7°C.

The truffle monitoring sites have not generally experienced anomalies of this magnitude over the past 40 years—with the exception of the record‐breaking 2003 European summer heat wave (Figure 2e). However, the 40‐year climate record includes increasingly frequent 1 and 2°C temperature anomalies, particularly in the last decade. Thus, during the 30‐year period from 1980 to 2010, +1°C anomalies took place once a decade and +2°C anomalies once every 30 years; for the most recent period (2011–2019), +2°C anomalies occurred every other year.

### 3.2 Summary of raw monitoring results

**FIGURE 4:**
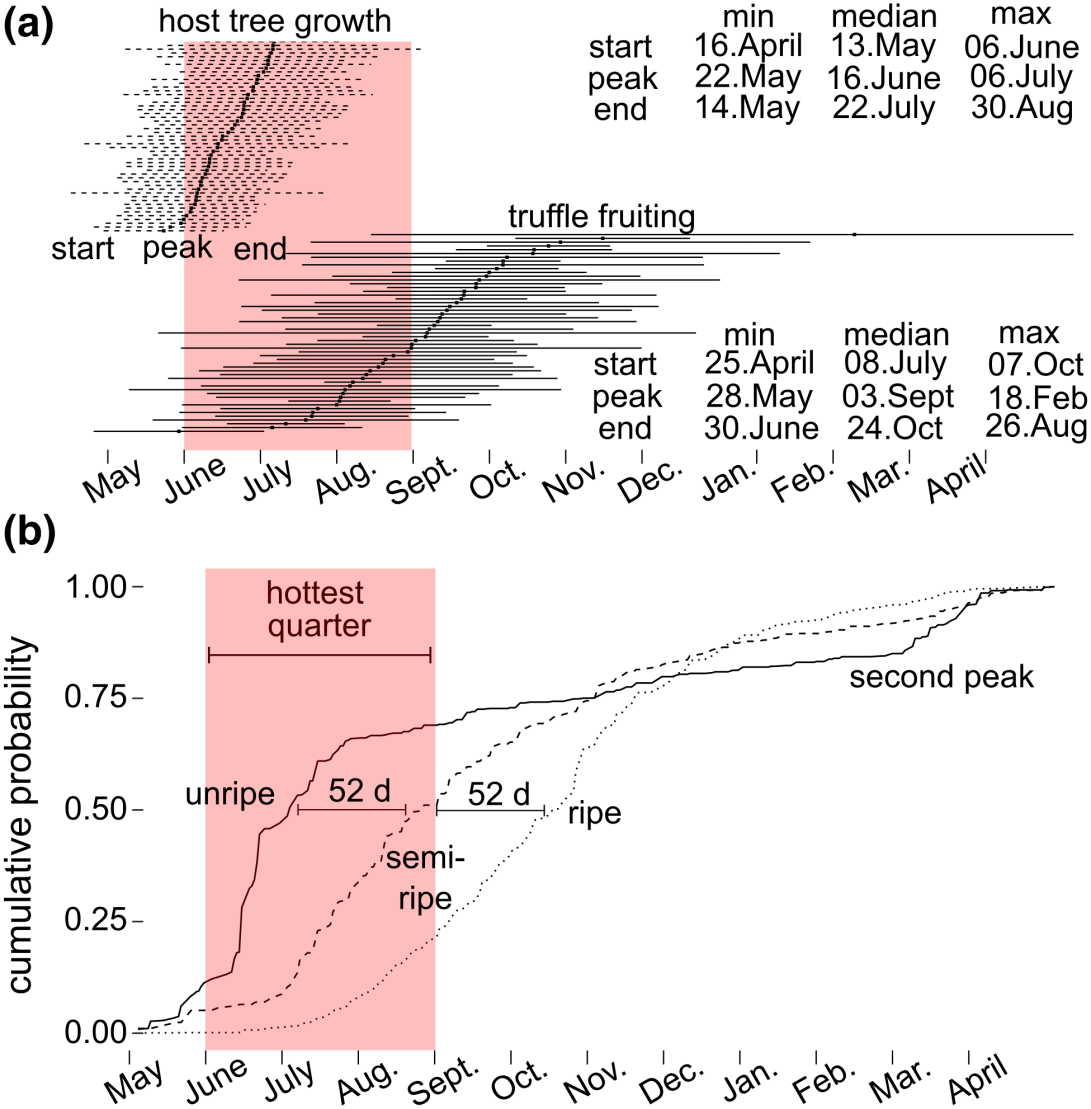
The hottest quarter of the year (red bars) occurs over the peak and latter half of host‐tree growing season and beginning of the (more variable) truffle fruiting season. (a) The seasonality of host‐tree growth and truffle fruiting arranged on the y‐axis by category (tree vs. truffle) and increasing peak date. (b) The cumulative probability for the collection of unripe, semi‐ripe, and ripe‐truffles.

Over the course of 1781 independent truffle surveys, citizen scientists sampled a total of 6633 *T. aestivum* fruitbodies (with a total mass over 100 kg) over the 20 sites from the period of 2011–2018. The timing of truffle fruiting varied among sites and years. According to median values, fruiting began in early July, reached peak productivity in September, and ended late October. However, there was considerable variability among sites: fruiting start was observed as early as April 22 and as late as June 30 (Figure 4a). Unripe truffles were the first observed, with a peak in mid‐June, followed 52 days later by a peak in ripe truffles and 52 days after this with a peak in over‐ripe truffles. However, whereas unripe truffles emerged first, they continued to emerge continuously during the fruiting season, and exhibited a second peak in early spring (Figure 4b).

The *T. aestivum* fruiting season overlaps with the second half of the tree growing season, which also corresponds to the hottest quarter of the year (Figures 3a,b and 4a). Tree growth starts earlier than truffle fruiting, generally beginning in the spring (median May 13), with all trees reaching their peak growth rate before July 8 (before the majority of truffle sites begin their fruiting). The second half of the tree growing season spans from (median values) 16 June to 22 July, peaking and ending during the hottest quarter of the year. Relative to *T. aestivum* fruiting season, tree growing seasons had 73.1% less variability in length (the standard deviation of the truffle fruiting and tree growing seasons were 59.5 and 16.0 days, respectively).

Over the entire monitoring period, the Bb site had the greatest total truffle mass (g), accounting for ~19.3% of the mass and truffle numbers harvested during the study. However, when total mass is divided by the area (hereafter yield, g m^−2^) of the monitoring plot, the most productive site was Sl (which, relative to Bb, produced 53.8% as much truffle fruitbody mass over an area 2.5% as large, Table 1).

### 3.3 Variance decomposition

Variability in truffle yield among sites and years was related primarily to differences in the number of truffles sampled and less so to the mean truffle mass. There was a four‐fold greater range of values over the log(truffles m^−2^) relative to log(mean truffle mass) plotted for each year. Variance decomposition revealed that 82.7% of the variance in log(yield) is explained by its covariance with log(truffle number), whereas the remaining 17.2% is explained by its covariance with log(mean truffle mass) (Figure S4).

When variability in total truffle mass is decomposed into among‐ and within‐site components, it reveals that approximately 45% of variability exists among site (SS_site_/SS_total_) and the remaining 55% of variability is due to the residual (interannual) variability within each site (SS_res_/SS_total_).

### 3.4 Linear mixed‐effects model with climate

**FIGURE 5:**
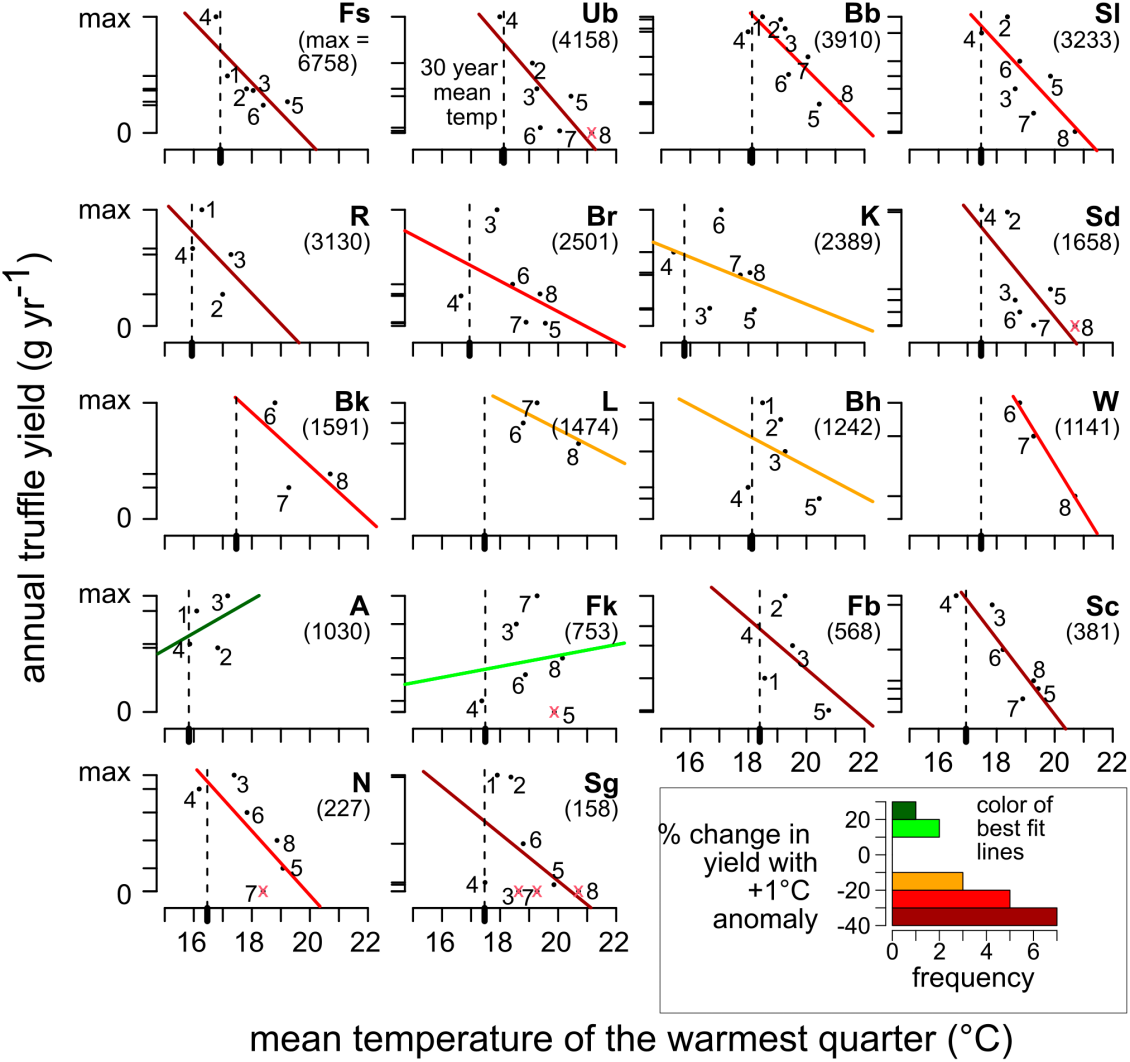
Annual truffle yield of each site in gram (*y*‐axis) tends to decline with the mean temperature of the warmest quarter in °C (*x*‐axis). The 30‐year (1980–2010) mean temperature of the hottest quarter is indicated by the broken line. Sites are ordered according to the maximum observed productivity. Data points are labeled by year (2011–2018). Red x's indicate yields of zero. Best‐fit linear regression lines are colored according to the predicted % change in yield given a one degree increase in temperature

Mixed‐effect models demonstrate a statistically significant decline in annual truffle productivity (kg year^−1^) with increases in the mean temperature of the hottest quarter when monitoring sites have a random slope and intercept (*p* < .001, df = 66.55, *t* = −4.47). Every increase of 1°C in mean summer temperature is associated with approximately 408.76 ± 91.54 (STE) g reduction in truffle yield across sites (*R* ^2^_conditional_ = 0.734, *R* ^2^_marginal_ = 0.175). For each site, we calculated the predicted % change in yield with a 1°C temperature anomaly relative to the site's 30‐year average summer temperature. The predicted changes range from −70.8% (Sl) to 20.6% (A, with only one other site, Fk, having a positive predicted change in yield with temperature of the hottest quarter), with a mean and median value of −21.6% and −21.8%, respectively (Figure 5).

### 3.5 Random forests and truffle yields

**FIGURE 6:**
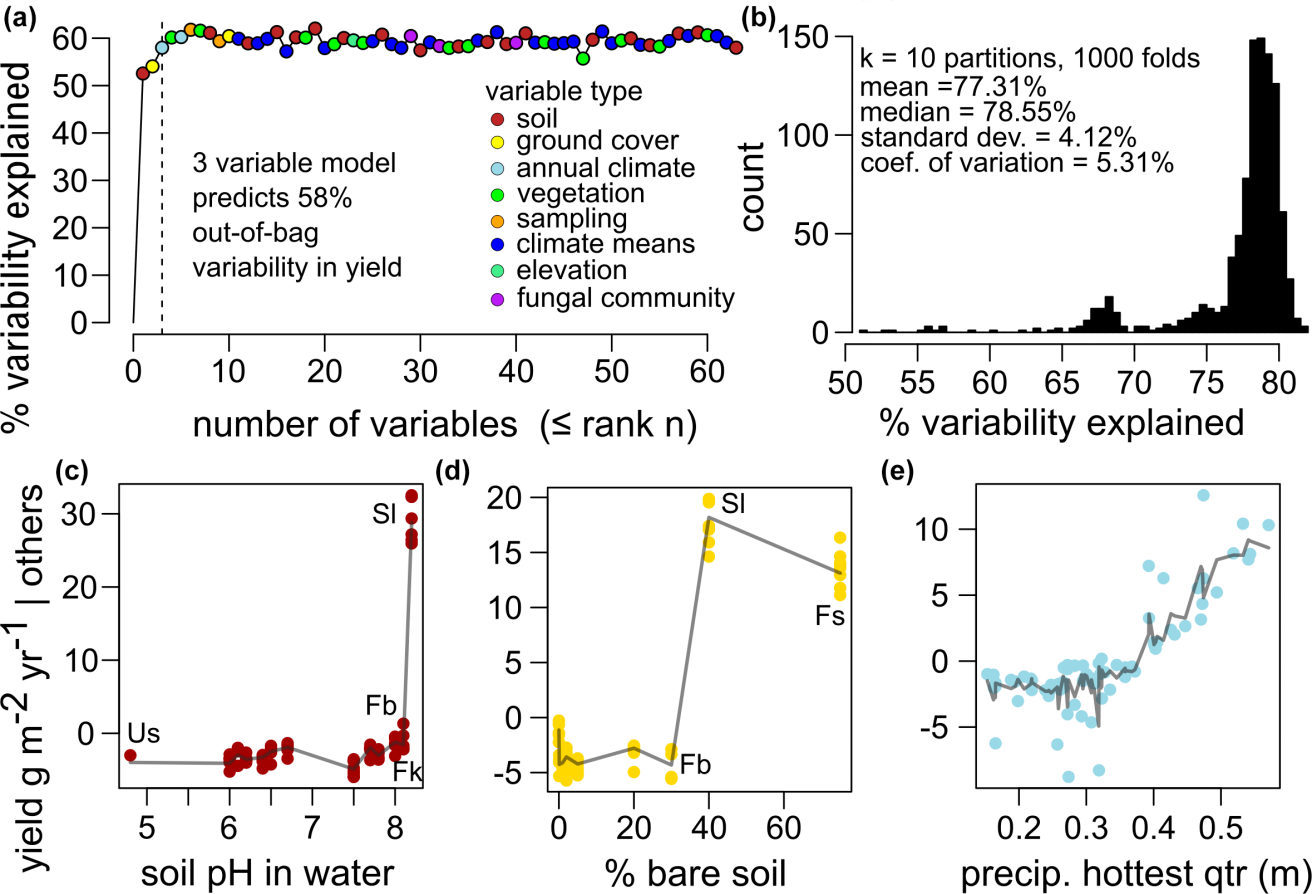
The majority of inter‐site and inter‐annual variability truffle yields can be stably accounted for using only three predictor variables. (a) The % out‐of‐bag variability explained in truffle yield (g m^−2^) by random forest models versus the number of predictors (sorted by importance, Table S1). (b) The distribution of coefficient of determination for 1000 iterations of *k* = 10‐fold cross‐validation. (c,d, and e) Partial feature contributions of the three most important predictors of truffle yield, showing highest yield among sites with (c) alkaline pH, (d) greater % of bare soil, and (e) greater total precipitation during the hottest quarter.

A random forest model explains approximately 58% of out‐of‐bag variability in annual yields with three variables (ranked from most‐to‐least important): soil pH, % bare soil (land surface not covered by herbs, moss, and/or litter), and precipitation sum of the warmest quarter (Figure 6a,c–e). *k*‐fold cross‐validation reveals that model performance with only the three most important variables is stable to a 10% loss of training data—the mean coefficient of determination (c.o.d.) of the 1000 k‐fold models was 77.31% with an among‐model standard deviation of 4.12% and coefficient of variation of 5.31% (Figure 6b). Inclusion of other variables, including other soil physicochemical, vegetative community and soil fungal meta‐community principal component (PC) axes increase model complexity but offer no additional predictive power (Figure 6a; Table S1).

Partial feature contributions reveal that truffle yields are highest among sites with alkaline soils and a high percentage of bare soil, with steep inflection points at pH > 8.1 (explaining the high yield of the most productive site, Sl) and % of bare soils >40% (Figure 6c,d). The third variable, precipitation sum of the hottest quarter, explains both among‐ and within‐site variability in annual truffle yield. The partial feature contribution shows yields increase with precipitation, particularly at >300 mm (Figure 6e).

### 3.6 Negative temperature versus precipitation correlations during the hottest quarter

The linear mixed‐effect models identify temperature as more important than precipitation for total truffle productivity within a site (g year^−1^), whereas the random forest models identify precipitation as somewhat more important than temperature for truffle yields (g m^−2^ year^−1^) among sites and years (Table S1). However, precipitation and temperature during the hottest quarter are not independent of one another: each 100 mm increase in precipitation is associated with a 1° drop in temperature (Figure S3). Thus, both models track the decline of *T. aestivum* fruiting in hot, dry years.

### 3.7 Truffle productivity and dendrometer data

**FIGURE 7:**
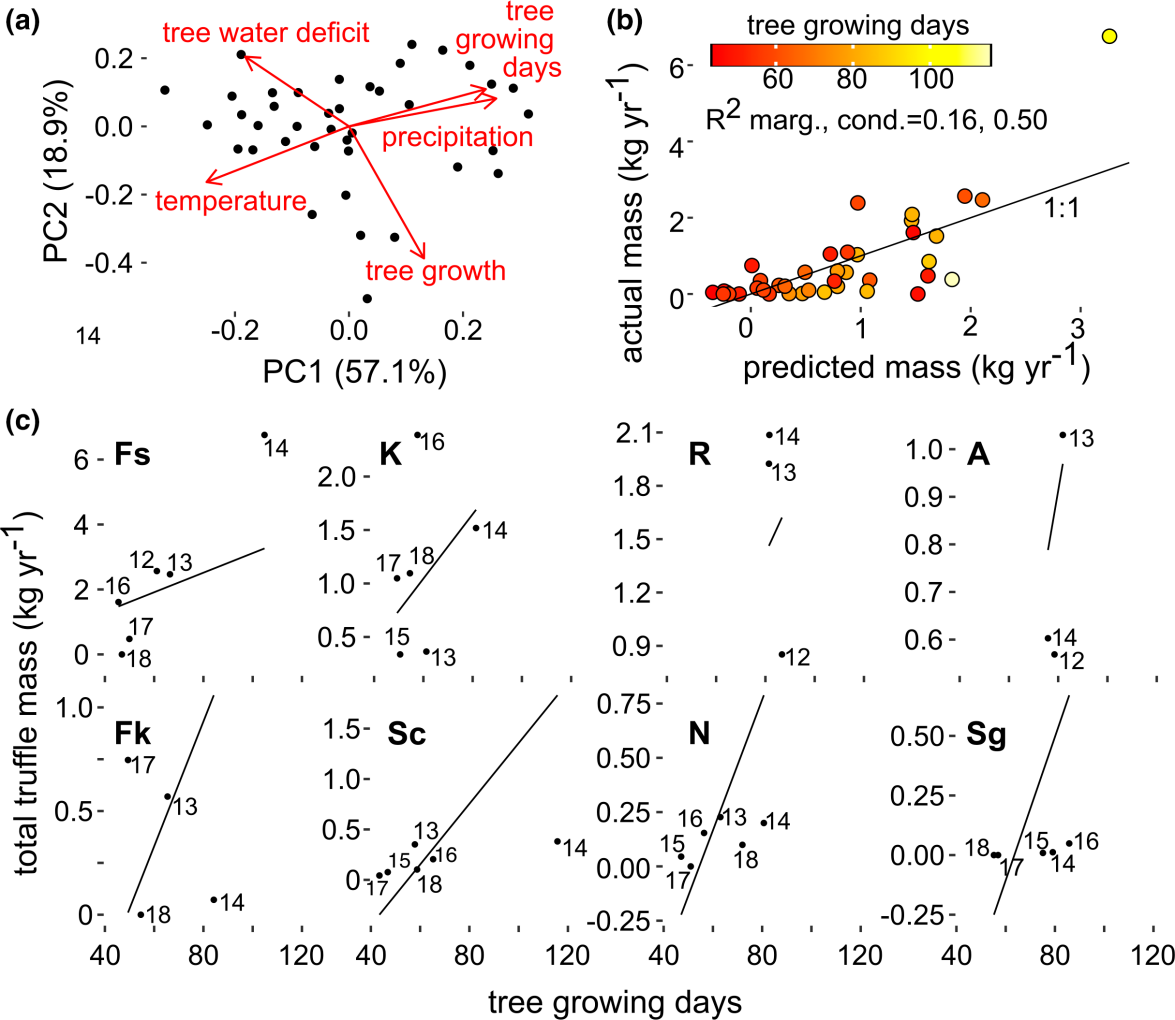
Hot and dry summers reduce the length of the tree growing season, resulting in reduced truffle yields. (a) The orthogonal vectors of climate and dendrology data for the subset of eight sites with available data. (b) The actual versus predicted truffle mass using a linear mixed effects model with a random intercept for each site. (c) The relationship between tree growing season length and truffle mass for each site. The lines show the associated prediction (the appearance of different slopes is caused by differences in scale of the *y*‐axis among sites).

To visualize the relationship between climate and dendrological measurements, we scaled these variables and performed a PC analysis. The first two PC axes explained 71.60% (PC1 = 51.48%, PC2 = 20.12%) of the orthogonal variability among sites and years. Visual inspection of the loading values on the first PC axis shows that tree growing season is negatively correlated with temperature and positively correlated with precipitation (both values calculated during the hottest quarter of the year). Likewise, inspection of the second PC axis shows that tree growth is negatively correlated with tree water deficit (Figure 7a).

As dendrological data were available for only a subset of sites (Table 1), we fit a separate linear mixed‐effects models for the effects of tree growth and water deficit variables on total truffle mass. We found that the total truffle mass (g year^−1^) increases with the length of the tree growing season (*p* = .006, df = 32.59, *t* = 2.958). For each one‐day increase in tree growing season, there was an 22.60 ± 7.64 g increase in truffle mass (estimate ± standard error, *R* ^2^_conditional_ = 0.690, *R* ^2^_marginal_ = 0.084). In contrast, there was no statistically significant relationship between annual truffle mass (g year^−1^) and total annual tree growth or mean water deficit.

## 4 DISCUSSION

Our results show that *T. aestivum* populations in the center of the species' broad European climatic range produce less fruitbodies during hot, dry summers. Our linear models predict that anomalies of 2–3°C are sufficient to stop truffle productivity among our monitored sites. Based on first approximation of the climatic niche, which is commonly inferred from species‐distribution records, these monitored truffle populations should be far from the hot‐edge of their climatic envelope. Our findings suggest that this approximation is erroneous. Instead, *T. aestivum* populations appear locally adapted to narrow climate conditions, sensitizing them to the expected increase in summer drought frequency throughout the 21st century.

The hottest year observed over the monitoring period was insufficient to push any one of our 20 truffle sites outside the 30‐year average summer temperatures experienced by the populations at the hot, Mediterranean edge of the species distribution (Figure 2a–c). Summer truffle populations in Spain and Italy produce fruitbodies when 30‐year summer temperatures average 20°C, whereas populations in the cooler forests of Switzerland and Southwest Germany do not fruit when summer temperatures reach 20°C in a single year. Summer truffles exhibit the same pattern as European beech trees and marine copepods, which have been shown to be sensitive to climate changes, even when they are insufficient to push them to the edge of the respective species' environmental envelope (Gárate‐Escamilla et al., 2019; Kelly et al., 2012; Knutzen et al., 2017).

Truffles depend on animals to eat their fruitbodies and distribute their spores, which are most likely needed as fertilizing partner to produce fruitbodies (Selosse et al., 2017). Thus, sustained fruitbody removal could result in local spore limitation and a consequent decline in truffle productivity. However, the effects of sampling were minor compared to the effects of interannual temperature and precipitation (in both linear mixed effects and random forest models). The stronger association of truffle mass with interannual climate is best exemplified by data from year 2014—three years into the sampling. Summer 2014 was unique during the monitoring period in having lower temperature and higher precipitation than the 30‐year average for each site. These conditions also led to increased fruitbody production, particularly among the sites that suffered the largest interannual declines over the entire monitoring period. Even if harvesting reduces an inoculation source, a previous study showed that a high percentage of mature fruitbodies are not detected by trained truffle dogs (Schneider‐Maunoury et al., 2019). The fall of the summer truffle production is associated with a heating and drying trend expected to continue and intensify with global climate change (Christidis et al., 2015; Stott et al., 2004).

Our data do not address whether declines fruitbody production are associated with a decline in ectomycorrhizal formation, spore germination, failure to form primordia, or to a decline in hyphal mass. Among wood‐decaying fungi, fruitbody production and mycelial abundance are correlated with one another (Ovaskainen et al., 2013), the same was found for diverse ectomycorrhizal fungal species (Guidot et al., 2004; Lian et al., 2006; Zhou et al., 2001). However, several studies comparing fruitbody presence and abundance with belowground structures such as ectomycorrhizal root tips or mycelia show incongruence (Anderson & Cairney, 2007). Mycelium density and fruitbody production have been shown to be correlated in both the Périgord (Suz et al., 2008) and summer truffle (Todesco et al., 2019). By contrast, although mycelium density and fruity bodies are correlated in the congeneric white truffle (*T. magnatum*), mycorrhizal connections and hyphal mycelia have been detected in the absence of fruitbody production (Murat et al., 2005; Zampieri et al., 2009). This creates uncertainty as to whether, when *T. aestivum* fails to fruit, it continues to persist in soil/plant roots belowground.

Based on genotyping fruitbodies, *T. aestivum* individuals are mostly short‐lived, with a high annual turnover of genotypes (Molinier, Murat, et al., 2016). This implies that mycelia would also be rather short‐lived coinciding with fruitbody production as proven for the ruderal ectomycorrhizal fungal species *Hebeloma cylindrosporum* (Guidot et al., 2004). However, a few perennial truffle genotypes were also present and could potentially persist as mycelia without forming truffles. Thus, it is possible that sites with no fruitbodies contain resilient *T. aestivum* populations belowground. In fact, *T. aestivum* mycelial growth has proved to be drought resistant as compared to other fungal species, being able to grow at soil hydric potentials below the temporary wilting point (i.e., pF 4.2; Todesco et al., 2019).

It is therefore currently unclear whether populations that ceased fruiting during hot/dry years will recover in cooler/wetter ones. In this context, summer drought could be detrimental to the annual success of truffle fruitbody foragers without imperiling the viability of the truffle populations. However, the absence of truffle fruitbodies also indicates the absence of sexual recombination and progeny production in the form of spores. In evolutionary terms, sex generates new and potentially beneficial allele combinations that are the basis for local adaptation. Furthermore, truffle fruitbodies facilitate active dispersion and gene flow. Their absence could be especially problematic for local populations when environmental variation exceeds phenotypic plasticity of existing individuals.

Truffles may be adapted to local climates by their host trees. Thus, hot and dry summers that reduce host tree growth and photosynthesis (McDowell, 2011) could consequently reduce the carbon provisioned to mycorrhizal fungi—including *T. aestivum* (Hagedorn et al., 2016; Joseph et al., 2020). However, we found no relationship between truffle productivity and either total tree growth or tree water deficit during the hottest quarter. This might partly be explained by the finding that tree growth is more sensitive to atmospheric drought than carbon assimilation (Zweifel et al., 2021). Truffle fruitbody growth is directly dependent on recent assimilates during the early season and from stored carbon after the end of the host trees' carbon assimilation (Tacon et al., 2013). During the hottest quarter of the year, higher temperatures are associated with shorter tree growing seasons, lower precipitation, and decreased truffle productivity. While direct effects of these climatic parameters on truffle fruitbody production are likely, indirect effects via reduced supply of recent assimilates due to shortened growing seasons are conceivable as well.

Variability in annual truffle yield among our sites was associated with soil pH, % bare soils, and interannual precipitation totals during the hottest quarter—and not strongly associated with 60 other soil physicochemical, climate, and vegetative and soil fungal community variables (Table S1). Thus, *T. aestivum* yields were not influenced by soil nitrogen, texture, cation exchange capacity, site elevation, tree species, or soil fungal species composition, nor any of the 19 bioclimatic variables drawn from 30‐year climate means. In fact, we found that our random forest model performance could not be improved by adding a single additional variable over our top three (Figure 6a). Together, these results suggest that—at least within sites that met our criteria for truffle monitoring—truffle yields are governed by a few principal factors.

Our results for which factors are important are not surprising given previous studies. For example, soil pH was the most important predictor of *T. aestivum* fruitbody yields in our random forest models, which is consistent with previous studies linking *T. aestivum* to alkaline soils (Hilszczańska et al., 2019; Robin et al., 2016). Likewise, the congeneric Périgord truffle, *T. melanosporum* is known to suppress vegetation by releasing allelochemicals that produce characteristic brûlé patterns of bare soil around the stems of host trees (Taschen et al., 2020). These brûlés are thought to create a microenvironment conducive to fructification. Our results strongly corroborate these earlier findings regarding the importance of soil pH and % bare soils, as these two emerged as dominant explanatory variables in a completely inductive model selection procedure that could have assessed 2^63^ possible variable combinations.

An inspection of the partial feature contributions reveals the nonlinear, threshold responses of *T. aestivum* yields to environmental factors. *T. aestivum* yields are insensitive to soil pH until they increase sharply at pH > 8.1. Likewise, truffle yields increase sharply when bare soils reach 40% and then level off. However, these soil and land‐cover responses were driven by a few sites and therefore need to be verified including additional sites. Response to precipitation during the hottest quarter was also nonlinear, with little differences in *T. aestivum* yields among sites and years until precipitation >300 mm. The shape of yield responses is useful for predicting how populations will respond to climate change and for managing the productivity of truffle plantations. For example, our data suggest that irrigation and soil amendments to increase pH might have little effect on truffle yields unless they push populations beyond their response thresholds.

Based on restricted species occurrence to the southern parts in Europe, we would expect the Mediterranean Périgord truffle (*T. melanosporum*) to be more tolerant to hot and dry conditions than the more cosmopolitan summer truffle. Accordingly, we would predict that prolonged climate warming would lead to a replacement of *T. aestivum* along the species' southern, hot range‐limit with the more heat‐tolerant *T. melanosporum*. However, the opposite is the case. Whereas a northward migration is documented for the Périgord truffle (Büntgen et al., 2019), the summer truffle mycelia better resist hotter and particularly drier conditions (Piñuela et al., 2021) and seem to replace the Périgord truffle in wild sites on the Iberian Peninsula (Piñuela et al., 2021; Sánchez et al., 2016). The data presented here now suggest an additional dimension. If northern *T. aestivum* populations cannot tolerate temporal climate trends that fall within the species contemporary, spatial‐climate envelope, then there may be declines in *T. aestivum* well north of the migration frontier of *T. melanosporum*. Such climatic sensitization of *T. aestivum* could lead to prolonged absence of both *Tuber* species and their associated ecosystem services.

The sensitivity of *T. aestivum* populations observed here suggests that these central populations cannot tolerate temporal heating and drying trends similar to populations in southern Europe, or/and that they currently undergo stronger selection pressures than southern populations. Such sensitization puts *T. aestivum* in the company of common tree and crustacean species that also fail to tolerate climates falling inside the respective species' envelope (Kelly et al., 2012; Knutzen et al., 2017). Local adaptation could explain the sensitization of *T. aesitivum*. Within the true truffle genus, genetic strains of the congeneric *T. borchii* strains have been shown to have genetically different heat‐stress thresholds (Leonardi et al., 2017). Population genetic data indicate that *T. aestivum* shows limited gene flow, with strong population differentiation even if only 3 km apart (Molinier, Murat, et al., 2016). Within populations, significant isolation by distance indicates that offspring generally stay close to the parents (Molinier, Murat, et al., 2016) and inbreeding is likely as seen for the Périgord truffle (Selosse et al., 2017). Limited gene flow is characteristic for animal‐ and not wind‐dispersed, hypogeous fungi (Douhan et al., 2011). In addition, spatial genetic structure indicates that most spores do not travel at all but colonize the soil when undetected fruitbodies decay and release them (Molinier, Murat, et al., 2016; Selosse et al., 2017). Such limited gene flow indicates that populations may suffer from an adaptational lag to local climate conditions (Aitken et al., 2008). Thus, if environmental change happens too fast, then beneficial alleles from more heat‐tolerant southern populations may be out of reach.

A critical implication of the present study, combined with similar findings for other organisms, is that center‐of‐range populations are not safe from climate change. As global climate change increases both long‐term mean temperatures and the incidence of extreme weather anomalies—particular summer drought and heat waves—climate envelopes defined by species‐level occurrences may underestimate local responses to short‐term climate trends. Our results also call for genomic studies on local adaptation of these precious fungi, which could inform the application of climate‐adjusted provenances (Prober et al., 2015) for truffle cultivation and restoration as proposed for plant species.

## AUTHOR CONTRIBUTIONS

Simon Egli, Ulf Büntgen, Uli Stobbe, Ludger Sproll, Willy Tegel, and Martina Peter planned and coordinated the study, Simon Egli, Ulf Büntgen, Uli Stobbe, Willy Tegel, Ludger Sproll, and Istvan Bagi collected truffle data, Barbara Moser performed the vegetation analyses, Matthias Haeni and Roman Zweifel analyzed the dendrometer data, Virginie Molinier and Marc Buée collected and performed soil chemical and molecular fungal community analyses, Brian S. Steidinger conducted the statistical analyses and drafted the first manuscript. All authors provided important intellectual content and were involved in critically revising the manuscript.

## CONFLICT OF INTEREST

The authors declare they have no conflict of interest.

## Supporting information


Data S1

## Data Availability

All of the raw data and code required to repeat our analyses are archived in Dryad at https://doi.org/10.5061/dryad.zs7h44jd3 (Steidinger & Peter, 2022).

## References

[gcb16424-bib-0001] Aitken, S. N. , Yeaman, S. , Holliday, J. A. , Wang, T. , & Curtis‐McLane, S. (2008). Adaptation, migration or extirpation: Climate change outcomes for tree populations. Evolutionary Applications, 1(1), 95–111. 10.1111/j.1752-4571.2007.00013.x 25567494 PMC3352395

[gcb16424-bib-0002] Alexander, L. (2011). Extreme heat rooted in dry soils. Nature Geoscience, 4(1), 12–13. 10.1038/ngeo1045

[gcb16424-bib-0003] Anderson, I. C. , & Cairney, J. W. G. (2007). Ectomycorrhizal fungi: Exploring the mycelial frontier. FEMS Microbiology Reviews, 31(4), 388–406. 10.1111/j.1574-6976.2007.00073.x 17466031

[gcb16424-bib-0004] Araújo, M. B. , Whittaker, R. J. , Ladle, R. J. , & Erhard, M. (2005). Reducing uncertainty in projections of extinction risk from climate change. Global Ecology and Biogeography, 14(6), 529–538. 10.1111/j.1466-822X.2005.00182.x

[gcb16424-bib-0005] Atkins, K. E. , & Travis, J. M. J. (2010). Local adaptation and the evolution of species' ranges under climate change. Journal of Theoretical Biology, 266(3), 449–457. 10.1016/j.jtbi.2010.07.014 20654630

[gcb16424-bib-0006] Austin, M. (2007). Species distribution models and ecological theory: A critical assessment and some possible new approaches. Ecological Modelling, 200(1), 1–19. 10.1016/j.ecolmodel.2006.07.005

[gcb16424-bib-0007] Baragatti, M. , Grollemund, P.‐M. , Montpied, P. , Dupouey, J.‐L. , Gravier, J. , Murat, C. , & Le Tacon, F. (2019). Influence of annual climatic variations, climate changes, and sociological factors on the production of the Périgord black truffle (*Tuber melanosporum* Vittad.) from 1903–1904 to 1988–1989 in the Vaucluse (France). Mycorrhiza, 29(2), 113–125. 10.1007/s00572-018-0877-1 30603794

[gcb16424-bib-0008] Bates, D. , Mächler, M. , Bolker, B. , & Walker, S. (2015). Fitting linear mixed‐effects models using lme4. Journal of Statistical Software, 67(1), 1–48. 10.18637/jss.v067.i01

[gcb16424-bib-0009] Beaumont, L. J. , Hughes, L. , & Poulsen, M. (2005). Predicting species distributions: Use of climatic parameters in BIOCLIM and its impact on predictions of species' current and future distributions. Ecological Modelling, 186(2), 251–270. 10.1016/j.ecolmodel.2005.01.030

[gcb16424-bib-0010] Benucci, G. M. N. , Raggi, L. , Albertini, E. , Grebenc, T. , Bencivenga, M. , Falcinelli, M. , & Di Massimo, G. (2011). Ectomycorrhizal communities in a productive *Tuber aestivum* Vittad. Orchard: Composition, host influence and species replacement. FEMS Microbiology Ecology, 76(1), 170–184. 10.1111/j.1574-6941.2010.01039.x 21223332

[gcb16424-bib-0011] Büntgen, U. , Egli, S. , Camarero, J. J. , Fischer, E. M. , Stobbe, U. , Kauserud, H. , Tegel, W. , Sproll, L. , & Stenseth, N. C. (2012). Drought‐induced decline in Mediterranean truffle harvest. Nature Climate Change, 2(12), 827–829. 10.1038/nclimate1733

[gcb16424-bib-0012] Büntgen, U. , Lendorff, H. , Lendorff, A. , Leuchtmann, A. , Peter, M. , Bagi, I. , & Egli, S. (2019). Truffles on the move. Frontiers in Ecology and the Environment, 17(4), 200–202. 10.1002/fee.2033

[gcb16424-bib-0013] Büntgen, U. , Tegel, W. , Egli, S. , Stobbe, U. , Sproll, L. , & Stenseth, N. C. (2011). Truffles and climate change. Frontiers in Ecology and the Environment, 9(3), 150–151. 10.1890/11.WB.004

[gcb16424-bib-0014] Čejka, T. , Trnka, M. , Krusic, P. J. , Stobbe, U. , Oliach, D. , Václavík, T. , Tegel, W. , & Büntgen, U. (2020). Predicted climate change will increase the truffle cultivation potential in Central Europe. Scientific Reports, 10(1), 21281. 10.1038/s41598-020-76177-0 33277535 PMC7719165

[gcb16424-bib-0015] Chen, I.‐C. , Hill, J. K. , Ohlemüller, R. , Roy, D. B. , & Thomas, C. D. (2011). Rapid range shifts of species associated with high levels of climate warming. Science, 333(6045), 1024–1026. 10.1126/science.1206432 21852500

[gcb16424-bib-0016] Christidis, N. , Jones, G. S. , & Stott, P. A. (2015). Dramatically increasing chance of extremely hot summers since the 2003 European heatwave. Nature Climate Change, 5(1), 46–50. 10.1038/nclimate2468

[gcb16424-bib-0017] Copernicus Climate Change Service . (2019). ERA5‐land hourly data from 2001 to present [data set]. ECMWF. 10.24381/CDS.E2161BAC

[gcb16424-bib-0018] Čufar, K. , Prislan, P. , de Luis, M. , & Gričar, J. (2008). Tree‐ring variation, wood formation and phenology of beech (*Fagus sylvatica*) from a representative site in Slovenia, SE Central Europe. Trees, 22(6), 749–758. 10.1007/s00468-008-0235-6

[gcb16424-bib-0019] Diez, J. , Kauserud, H. , Andrew, C. , Heegaard, E. , Krisai‐Greilhuber, I. , Senn‐Irlet, B. , Høiland, K. , Egli, S. , & Büntgen, U. (2020). Altitudinal upwards shifts in fungal fruiting in the Alps. Proceedings of the Royal Society B: Biological Sciences, 287(1919), 20192348. 10.1098/rspb.2019.2348 PMC7015340 31964234

[gcb16424-bib-0020] Dongmo, M. A. K. , Hanna, R. , Smith, T. B. , Fiaboe, K. K. M. , Fomena, A. , & Bonebrake, T. C. (2021). Local adaptation in thermal tolerance for a tropical butterfly across ecotone and rainforest habitats. Biology Open, 10(4), bio058619. 10.1242/bio.058619 34416009 PMC8053492

[gcb16424-bib-0021] Douhan, G. W. , Vincenot, L. , Gryta, H. , & Selosse, M.‐A. (2011). Population genetics of ectomycorrhizal fungi: From current knowledge to emerging directions. Fungal Biology, 115(7), 569–597. 10.1016/j.funbio.2011.03.005 21724164

[gcb16424-bib-0022] Dyderski, M. K. , Paź, S. , Frelich, L. E. , & Jagodziński, A. M. (2018). How much does climate change threaten European forest tree species distributions? Global Change Biology, 24(3), 1150–1163. 10.1111/gcb.13925 28991410

[gcb16424-bib-0023] Ebenman, B. , & Jonsson, T. (2005). Using community viability analysis to identify fragile systems and keystone species. Trends in Ecology and Evolution, 20(10), 568–575. 10.1016/j.tree.2005.06.011 16701436

[gcb16424-bib-0024] Elith, J. , Phillips, S. J. , Hastie, T. , Dudík, M. , Chee, Y. E. , & Yates, C. J. (2011). A statistical explanation of MaxEnt for ecologists. Diversity and Distributions, 17(1), 43–57. 10.1111/j.1472-4642.2010.00725.x

[gcb16424-bib-0025] Fick, S. E. , & Hijmans, R. J. (2017). WorldClim 2: New 1‐km spatial resolution climate surfaces for global land areas. International Journal of Climatology, 37(12), 4302–4315. 10.1002/joc.5086

[gcb16424-bib-0026] Gárate‐Escamilla, H. , Hampe, A. , Vizcaíno‐Palomar, N. , Robson, T. M. , & Benito Garzón, M. (2019). Range‐wide variation in local adaptation and phenotypic plasticity of fitness‐related traits in *Fagus sylvatica* and their implications under climate change. Global Ecology and Biogeography, 28(9), 1336–1350. 10.1111/geb.12936

[gcb16424-bib-0027] Grinnell, J. (1914). An account of the mammals and birds of the lower Colorado Valley: With especial reference to the distributional problems presented (classic reprint). Fb&c Limited.

[gcb16424-bib-0028] Gryndler, M. , Černá, L. , Bukovská, P. , Hršelová, H. , & Jansa, J. (2014). *Tuber aestivum* association with non‐host roots. Mycorrhiza, 24(8), 603–610. 10.1007/s00572-014-0580-9 24756631

[gcb16424-bib-0029] Guidot, A. , Debaud, J.‐C. , Effosse, A. , & Marmeisse, R. (2004). Below‐ground distribution and persistence of an ectomycorrhizal fungus. New Phytologist, 161(2), 539–547. 10.1046/j.1469-8137.2003.00945.x 33873517

[gcb16424-bib-0030] Hagedorn, F. , Joseph, J. , Peter, M. , Luster, J. , Pritsch, K. , Geppert, U. , Kerner, R. , Molinier, V. , Egli, S. , Schaub, M. , Liu, J.‐F. , Li, M. , Sever, K. , Weiler, M. , Siegwolf, R. T. W. , Gessler, A. , & Arend, M. (2016). Recovery of trees from drought depends on belowground sink control. Nature Plants, 2(8), 1–5. 10.1038/nplants.2016.111 27428669

[gcb16424-bib-0031] Harrison, P. A. , Berry, P. M. , Butt, N. , & New, M. (2006). Modelling climate change impacts on species' distributions at the European scale: Implications for conservation policy. Environmental Science and Policy, 9(2), 116–128. 10.1016/j.envsci.2005.11.003

[gcb16424-bib-0032] Hersbach, H. , Bell, B. , Berrisford, P. , Hirahara, S. , Horányi, A. , Muñoz‐Sabater, J. , Nicolas, J. , Peubey, C. , Radu, R. , Schepers, D. , Simmons, A. , Soci, C. , Abdalla, S. , Abellan, X. , Balsamo, G. , Bechtold, P. , Biavati, G. , Bidlot, J. , Bonavita, M. , … Thépaut, J.‐N. (2020). The ERA5 global reanalysis. Quarterly Journal of the Royal Meteorological Society, 146(730), 1999–2049. 10.1002/qj.3803

[gcb16424-bib-0033] Hilszczańska, D. , Szmidla, H. , Sikora, K. , & Rosa‐Gruszecka, A. (2019). Soil properties conducive to the formation of *Tuber aestivum* Vitt. Fruiting bodies. Polish Journal of Environmental Studies, 28(3), 1713–1718. 10.15244/pjoes/89588

[gcb16424-bib-0034] Hutchinson, G. E. (1959). Homage to Santa Rosalia or why are there so many kinds of animals? The American Naturalist, 93(870), 145–159. 10.1086/282070

[gcb16424-bib-0035] Joseph, J. , Gao, D. , Backes, B. , Bloch, C. , Brunner, I. , Gleixner, G. , Haeni, M. , Hartmann, H. , Hoch, G. , Hug, C. , Kahmen, A. , Lehmann, M. M. , Li, M.‐H. , Luster, J. , Peter, M. , Poll, C. , Rigling, A. , Rissanen, K. A. , Ruehr, N. K. , … Gessler, A. (2020). Rhizosphere activity in an old‐growth forest reacts rapidly to changes in soil moisture and shapes whole‐tree carbon allocation. Proceedings of the National Academy of Sciences of the United States of America, 117(40), 24885–24892. 10.1073/pnas.2014084117 32958662 PMC7547207

[gcb16424-bib-0036] Kane, K. , Debinski, D. M. , Anderson, C. , Scasta, J. D. , Engle, D. M. , & Miller, J. R. (2017). Using regional climate projections to guide grassland community restoration in the face of climate change. Frontiers in Plant Science, 8, 1–11. https://www.frontiersin.org/article/10.3389/fpls.2017.00730 28536591 10.3389/fpls.2017.00730 PMC5422548

[gcb16424-bib-0037] Kaspari, M. , Clay, N. A. , Lucas, J. , Yanoviak, S. P. , & Kay, A. (2015). Thermal adaptation generates a diversity of thermal limits in a rainforest ant community. Global Change Biology, 21(3), 1092–1102. 10.1111/gcb.12750 25242246

[gcb16424-bib-0038] Kelly, M. W. , Sanford, E. , & Grosberg, R. K. (2012). Limited potential for adaptation to climate change in a broadly distributed marine crustacean. Proceedings of the Royal Society B: Biological Sciences, 279(1727), 349–356. 10.1098/rspb.2011.0542 PMC3223665 21653591

[gcb16424-bib-0039] Knutzen, F. , Dulamsuren, C. , Meier, I. C. , & Leuschner, C. (2017). Recent climate warming‐related growth decline impairs European beech in the center of its distribution range. Ecosystems, 20(8), 1494–1511. 10.1007/s10021-017-0128-x

[gcb16424-bib-0040] Lenoir, J. , & Svenning, J.‐C. (2015). Climate‐related range shifts–A global multidimensional synthesis and new research directions. Ecography, 38(1), 15–28. 10.1111/ecog.00967

[gcb16424-bib-0041] Leonardi, P. , Iotti, M. , Donati Zeppa, S. , Lancellotti, E. , Amicucci, A. , & Zambonelli, A. (2017). Morphological and functional changes in mycelium and mycorrhizas of *Tuber borchii* due to heat stress. Fungal Ecology, 29, 20–29. 10.1016/j.funeco.2017.05.003

[gcb16424-bib-0042] Lian, C. , Narimatsu, M. , Nara, K. , & Hogetsu, T. (2006). Tricholoma matsutake in a natural *Pinus densiflora* forest: Correspondence between above‐ and below‐ground genets, association with multiple host trees and alteration of existing ectomycorrhizal communities. New Phytologist, 171(4), 825–836. 10.1111/j.1469-8137.2006.01801.x 16918553

[gcb16424-bib-0043] Liaw, A. , & Wiener, M. (2002). Classification and regression by randomForest. R News, 2(3), 18–22.

[gcb16424-bib-0044] Matsuki, K. , Kuperman, V. , & Dyke, J. A. V. (2016). The random forests statistical technique: An examination of its value for the study of reading. Scientific Studies of Reading, 20(1), 20–33. 10.1080/10888438.2015.1107073 26770056 PMC4710485

[gcb16424-bib-0045] McDowell, N. G. (2011). Mechanisms linking drought, hydraulics, carbon metabolism, and vegetation mortality. Plant Physiology, 155(3), 1051–1059. 10.1104/pp.110.170704 21239620 PMC3046567

[gcb16424-bib-0046] Molinier, V. , Murat, C. , Baltensweiler, A. , Büntgen, U. , Martin, F. , Meier, B. , Moser, B. , Sproll, L. , Stobbe, U. , Tegel, W. , Egli, S. , & Peter, M. (2016). Fine‐scale genetic structure of natural *Tuber aestivum* sites in southern Germany. Mycorrhiza, 26(8), 895–907. 10.1007/s00572-016-0719-y 27460217

[gcb16424-bib-0047] Molinier, V. , Peter, M. , Stobbe, U. , & Egli, S. (2016). The Burgundy truffle (*Tuber aestivum* syn. Uncinatum): A truffle species with a wide habitat range over Europe. In A. Zambonelli , M. Iotti , & C. Murat (Eds.), True truffle (tuber spp.) in the world: Soil ecology, systematics and biochemistry (pp. 33–47). Springer International Publishing. 10.1007/978-3-319-31436-5_3

[gcb16424-bib-0048] Murat, C. , Vizzini, A. , Bonfante, P. , & Mello, A. (2005). Morphological and molecular typing of the below‐ground fungal community in a natural tuber magnatum truffle‐ground. FEMS Microbiology Letters, 245(2), 307–313. 10.1016/j.femsle.2005.03.019 15837387

[gcb16424-bib-0049] Nadeau, C. P. , Urban, M. C. , & Bridle, J. R. (2017). Climates past, present, and yet‐to‐come shape climate change vulnerabilities. Trends in Ecology and Evolution, 32(10), 786–800. 10.1016/j.tree.2017.07.012 28844791

[gcb16424-bib-0050] Ovaskainen, O. , Schigel, D. , Ali‐Kovero, H. , Auvinen, P. , Paulin, L. , Nordén, B. , & Nordén, J. (2013). Combining high‐throughput sequencing with fruit body surveys reveals contrasting life‐history strategies in fungi. The ISME Journal, 7(9), 1696–1709. 10.1038/ismej.2013.61 23575372 PMC3749500

[gcb16424-bib-0051] Pearson, R. G. , & Dawson, T. P. (2003). Predicting the impacts of climate change on the distribution of species: Are bioclimate envelope models useful? Global Ecology and Biogeography, 12(5), 361–371. 10.1046/j.1466-822X.2003.00042.x

[gcb16424-bib-0052] Piñuela, Y. , Alday, J. G. , Oliach, D. , Castaño, C. , Bolaño, F. , Colinas, C. , & Bonet, J. A. (2021). White mulch and irrigation increase black truffle soil mycelium when competing with summer truffle in young truffle orchards. Mycorrhiza, 31(3), 371–382. 10.1007/s00572-020-01018-x 33515357

[gcb16424-bib-0053] Prober, S. , Byrne, M. , McLean, E. , Steane, D. , Potts, B. , Vaillancourt, R. , & Stock, W. (2015). Climate‐adjusted provenancing: A strategy for climate‐resilient ecological restoration. Frontiers in Ecology and Evolution, 3, 1–5. 10.3389/fevo.2015.00065

[gcb16424-bib-0054] Pucko, C. , Beckage, B. , Perkins, T. , & Keeton, W. S. (2011). Species shifts in response to climate change: Individual or shared responses? 1, 2. The Journal of the Torrey Botanical Society, 138(2), 156–176. 10.3159/TORREY-D-10-00011.1

[gcb16424-bib-0055] Reich, P. B. , Sendall, K. M. , Rice, K. , Rich, R. L. , Stefanski, A. , Hobbie, S. E. , & Montgomery, R. A. (2015). Geographic range predicts photosynthetic and growth response to warming in co‐occurring tree species. Nature Climate Change, 5(2), 148–152. 10.1038/nclimate2497

[gcb16424-bib-0056] Reyna, S. , & Garcia‐Barreda, S. (2014). Black truffle cultivation: A global reality. Forest Systems, 23(2), 317–328. 10.5424/fs/2014232-04771

[gcb16424-bib-0057] Reyna‐Domenech, S. , & García‐Barreda, S. (2009). European black truffle: Its potential role in agroforestry development in the marginal lands of Mediterranean Calcareous Mountains. In A. Rigueiro‐Rodróguez , J. McAdam , & M. R. Mosquera‐Losada (Eds.), Agroforestry in Europe: Current status and future prospects (pp. 295–317). Springer Netherlands. 10.1007/978-1-4020-8272-6_14

[gcb16424-bib-0058] Robin, C. , Goutal‐Pousse, N. , & Le Tacon, F. (2016). Soil characteristics for *Tuber aestivum* (syn. *T. uncinatum*). In A. Zambonelli , M. Iotti , & C. Murat (Eds.), True truffle (tuber spp.) in the world: Soil ecology, systematics and biochemistry (pp. 211–231). Springer International Publishing. 10.1007/978-3-319-31436-5_13

[gcb16424-bib-0059] RStudio Team . (2022). RStudio: Integrated development environment for R. RStudio, PBC. http://www.rstudio.com/

[gcb16424-bib-0060] Sánchez, S. , de Miguel, A. M. , Sáez, R. , Martín‐Santafé, M. , Águeda, B. , Barriuso, J. , García‐Barreda, S. , Salvador‐Alcalde, D. , & Reyna, S. (2016). Summer truffle in the Iberian Peninsula: Current status and crop potential. ITEA, 112(1), 20–33.

[gcb16424-bib-0061] Schneider‐Maunoury, L. , Taschen, E. , Richard, F. , & Selosse, M.‐A. (2019). Soil spore bank in tuber melanosporum: Up to 42% of fruitbodies remain unremoved in managed truffle grounds. Mycorrhiza, 29(6), 663–668. 10.1007/s00572-019-00912-3 31701214

[gcb16424-bib-0062] Selosse, M.‐A. , Schneider‐Maunoury, L. , Taschen, E. , Rousset, F. , & Richard, F. (2017). Black truffle, a hermaphrodite with forced unisexual behaviour. Trends in Microbiology, 25(10), 784–787. 10.1016/j.tim.2017.05.010 28622845

[gcb16424-bib-0063] Soberón, J. , & Nakamura, M. (2009). Niches and distributional areas: Concepts, methods, and assumptions. Proceedings of the National Academy of Sciences of the United States of America, 106(Suppl 2), 19644–19650. 10.1073/pnas.0901637106 19805041 PMC2780935

[gcb16424-bib-0064] Somero, G. N. (2010). The physiology of climate change: How potentials for acclimatization and genetic adaptation will determine ‘winners’ and ‘losers’. Journal of Experimental Biology, 213(6), 912–920. 10.1242/jeb.037473 20190116

[gcb16424-bib-0065] Steidinger, B. , & Peter, M. (2022). Data from: The fall of the summer truffle: Recurring hot, dry summers result in declining fruitbody production of Tuber aestivum in Central Europe. Dryad. 10.5061/dryad.zs7h44jd3 PMC9828532 36200354

[gcb16424-bib-0066] Steinbauer, M. J. , Grytnes, J.‐A. , Jurasinski, G. , Kulonen, A. , Lenoir, J. , Pauli, H. , Rixen, C. , Winkler, M. , Bardy‐Durchhalter, M. , Barni, E. , Bjorkman, A. D. , Breiner, F. T. , Burg, S. , Czortek, P. , Dawes, M. A. , Delimat, A. , Dullinger, S. , Erschbamer, B. , Felde, V. A. , … Wipf, S. (2018). Accelerated increase in plant species richness on mountain summits is linked to warming. Nature, 556(7700), 231–234. 10.1038/s41586-018-0005-6 29618821

[gcb16424-bib-0067] Stobbe, U. , Büntgen, U. , Sproll, L. , Tegel, W. , Egli, S. , & Fink, S. (2012). Spatial distribution and ecological variation of re‐discovered German truffle habitats. Fungal Ecology, 5(5), 591–599. 10.1016/j.funeco.2012.02.001

[gcb16424-bib-0068] Stobbe, U. , Egli, S. , Tegel, W. , Peter, M. , Sproll, L. , & Büntgen, U. (2013). Potential and limitations of *Burgundy truffle* cultivation. Applied Microbiology and Biotechnology, 97(12), 5215–5224. 10.1007/s00253-013-4956-0 23666478

[gcb16424-bib-0069] Stott, P. A. , Stone, D. A. , & Allen, M. R. (2004). Human contribution to the European heatwave of 2003. Nature, 432(7017), 610–614. 10.1038/nature03089 15577907

[gcb16424-bib-0070] Streiblová, E. , Gryndlerová, H. , & Gryndler, M. (2012). Truffle brûlé: An efficient fungal life strategy. FEMS Microbiology Ecology, 80(1), 1–8. 10.1111/j.1574-6941.2011.01283.x 22225520

[gcb16424-bib-0071] Strobl, C. , Malley, J. , & Tutz, G. (2009). An introduction to recursive partitioning: Rationale, application, and characteristics of classification and regression trees, bagging, and random forests. Psychological Methods, 14(4), 323–348. 10.1037/a0016973 19968396 PMC2927982

[gcb16424-bib-0072] Suz, L. M. , Martín, M. P. , Oliach, D. , Fischer, C. R. , & Colinas, C. (2008). Mycelial abundance and other factors related to truffle productivity in tuber melanosporum–Quercus ilex orchards. FEMS Microbiology Letters, 285(1), 72–78. 10.1111/j.1574-6968.2008.01213.x 18510558

[gcb16424-bib-0073] Tacon, F. L. , Zeller, B. , Plain, C. , Hossann, C. , Bréchet, C. , & Robin, C. (2013). Carbon transfer from the host to tuber melanosporum mycorrhizas and ascocarps followed using a 13C pulse‐labeling technique. PLoS ONE, 8(5), e64626. 10.1371/journal.pone.0064626 23741356 PMC3669392

[gcb16424-bib-0074] Taschen, E. , Sauve, M. , Vincent, B. , Parladé, J. , van Tuinen, D. , Aumeeruddy‐Thomas, Y. , Assenat, B. , Selosse, M.‐A. , & Richard, F. (2020). Insight into the truffle brûlé: Tripartite interactions between the black truffle (*Tuber melanosporum*), holm oak (*Quercus ilex*) and arbuscular mycorrhizal plants. Plant and Soil, 446(1), 577–594. 10.1007/s11104-019-04340-2

[gcb16424-bib-0075] Thomas, C. D. , Cameron, A. , Green, R. E. , Bakkenes, M. , Beaumont, L. J. , Collingham, Y. C. , Erasmus, B. F. N. , de Siqueira, M. F. , Grainger, A. , Hannah, L. , Hughes, L. , Huntley, B. , van Jaarsveld, A. S. , Midgley, G. F. , Miles, L. , Ortega‐Huerta, M. A. , Townsend Peterson, A. , Phillips, O. L. , & Williams, S. E. (2004). Extinction risk from climate change. Nature, 427(6970), 145–148. 10.1038/nature02121 14712274

[gcb16424-bib-0076] Todesco, F. , Belmondo, S. , Guignet, Y. , Laurent, L. , Fizzala, S. , Le Tacon, F. , & Murat, C. (2019). Soil temperature and hydric potential influences the monthly variations of soil *Tuber aestivum* DNA in a highly productive orchard. Scientific Reports, 9(1), 12964. 10.1038/s41598-019-49602-2 31506577 PMC6736833

[gcb16424-bib-0077] Wedén, C. , Danell, E. , & Tibell, L. (2005). Species recognition in the truffle genus tuber–The synonyms *Tuber aestivum* and tuber uncinatum. Environmental Microbiology, 7(10), 1535–1546. 10.1111/j.1462-2920.2005.00837.x 16156727

[gcb16424-bib-0078] Welling, S. H. , Refsgaard, H. H. F. , Brockhoff, P. B. , & Clemmensen, L. H. (2016). Forest floor visualizations of random forests. ArXiv:1605.09196 [cs, stat], 1–25. http://arxiv.org/abs/1605.09196

[gcb16424-bib-0079] Zampieri, E. , Mello, A. , Bonfante, P. , & Murat, C. (2009). PCR primers specific for the genus tuber reveal the presence of several truffle species in a truffle‐ground. FEMS Microbiology Letters, 297(1), 67–72. 10.1111/j.1574-6968.2009.01655.x 19519770

[gcb16424-bib-0080] Zhou, Z. , Miwa, M. , Matsuda, Y. , & Hogetsu, T. (2001). Spatial distribution of the subterranean mycelia and Ectomycorrhizae of *Suillus grevillei* genets. Journal of Plant Research, 114(2), 179–185. 10.1007/PL00013981

[gcb16424-bib-0081] Zizka, A. , Silvestro, D. , Andermann, T. , Azevedo, J. , Duarte Ritter, C. , Edler, D. , Farooq, H. , Herdean, A. , Ariza, M. , Scharn, R. , Svantesson, S. , Wengström, N. , Zizka, V. , & Antonelli, A. (2019). CoordinateCleaner: Standardized cleaning of occurrence records from biological collection databases. Methods in Ecology and Evolution, 10(5), 744–751. 10.1111/2041-210X.13152

[gcb16424-bib-0082] Zweifel, R. , Haeni, M. , Buchmann, N. , & Eugster, W. (2016). Are trees able to grow in periods of stem shrinkage? New Phytologist, 211(3), 839–849. 10.1111/nph.13995 27189708

[gcb16424-bib-0083] Zweifel, R. , Sterck, F. , Braun, S. , Buchmann, N. , Eugster, W. , Gessler, A. , Häni, M. , Peters, R. L. , Walthert, L. , Wilhelm, M. , Ziemińska, K. , & Etzold, S. (2021). Why trees grow at night. New Phytologist, 231(6), 2174–2185. 10.1111/nph.17552 34118158 PMC8457160

[gcb16424-bib-0084] Zweifel, R. , Zimmermann, L. , & Newbery, D. M. (2005). Modeling tree water deficit from microclimate: An approach to quantifying drought stress. Tree Physiology, 25(2), 147–156. 10.1093/treephys/25.2.147 15574396

