# Supplementary material for: The fall of the summer truffle: Recurring hot, dry summers result in declining fruitbody production of Tuber aestivum in Central Europe
Source: Glob Chang Biol. 2022 Oct 6;28(24):7376–90. doi: 10.1111/gcb.16424 (PMC9828532; doi:10.1111/gcb.16424)
Supplement: Supplementary file 1 — Data S1 [file GCB-28-7376-s001.pdf]

Title: The fall of the summer truffle: recurring hot, dry summers result in declining fruitbody production of *Tuber aestivum* in central Europe

Authors: Brian S. Steidinger, Ulf Büntgen, Uli Stobbe, Willy Tegel, Ludger Sproll, Matthias Haeni, Barbara Moser, Istvan Bagi, José-Antonio Bonet, Marc Buée, Benjamin Dauphin, Fernando Martínez-Peña, Virginie Molinier, Roman Zweifel, Simon Egli, Martina Peter

Supplemental Information

Vegetation, Soil Physicochemical, and Soil Fungal Meta-Community Analysis

At 16 of the 20 truffle sites, the vegetation, soil physicochemical and fungal meta-community variables were assessed in a 200 m^2 circle plot between June and August 2014. The plots centered on the area with the highest *T. aestivum* production. Percent and total cover of all vascular plant species was estimated in three plant height classes (herb layer 0–50 cm; shrub layer 51–500 cm; tree layer >500 m) as outlined in (Moser et al., 2017) and the percent cover of bare soil (i.e. ground cover) was also estimated. This equaled the soil surface of the circle plot not covered by vascular plants, mosses or litter in the herb layer.

In each of the same 16 sites, soil cores were taken from within the 200 m^2 vegetation survey area in November 2014. The area was subdivided in 4 quarters, 4 randomly distributed cores (10 cm deep and at least 2 m apart) were taken within each subdivision and pooled. The soil cores were 10 cm deep and 2–3 m separated each core. Soil cores were placed in the cold room (+4°C) for maximum one week before processing. Roots were collected from the 4 soil cores per subdivision, sieved (2 mm), and homogenized. From each homogenate (4 homogenates per monitoring site), three 2 ml tubes were filled for DNA analysis (Illumina) and tubes were frozen at -80°C. The rest of soil was dried for physicochemical analyses for which the four homogenates per monitoring site were pooled and sent to SADEF laboratories (Aspach-le-Bas, France; parameters assessed see Table S1).

Fungal DNA from the soil cores was extracted using the PowerSoil MOBIO DNA Isolation kit according to the manufacturer's instructions. DNA elution was 10-fold diluted for further analyses. The ITS2 barcode of fungal organisms was amplified using equimolar mixes of the primers ITS3 (Mix1_Fungi, Mix3_Sebacinales, Mix4_Glomeromycota, Mix5_Sordariales) and ITS4 (Mix1_Fungi, Mix4_Tulasnellaceae) as recommended by Tedersoo et al. (2015). Four PCRs per DNA-sample were carried out and pooled (2X51°C, 2x55°C annealing temp). PCR-amplified and AMPPure XP (Beckman Coulter) purified products were sent to Genome Quebec (McGill University) for MiSeq sequencing. Only forward (R1) reads were used (no pairing), since reverse reads showed low quality. In brief, the reads were first filtered and trimmed using the fastq_filter command of USEARCH from UPARSE (Edgar, 2013) and the ITS region sequences were then dereplicated to remove duplicated sequences using the derep_fulllength command of USEARCH. The dereplicated sequences were selected and the singletons were discarded. Operational taxonomic units (OTUs) were generated using USEARCH with a 97% similarity threshold and the taxonomic assignation was determined for each OTU representative sequence using the Basic Local Alignment Search Tool (BLAST) algorithm v 2.2.23 (Altschul et al., 1990) against the UNITE database release (https://unite.ut.ee/). After removing plant reads (the used primers also weakly amplify ITS regions of some plants) and one sample with low read numbers, samples were rarefied to 1,830 reads.

Sequences for each fungal OTU were first aggregated by site (taking the sum over all soil cores). All OTU singletons were removed. Sequences were rarefied to an even depth and converted to proportional abundances for each site. We characterized the orthogonal differences separately for vegetative (tree layer only) and fungal community using three separate principal component (PC) analyses each using the prcomp function in RStudio (v 1.1.463). These fungal and vegetative PC axes were included as potential predictor variables in our machine learning / model selection analyses of total fruitbody production among sites and years (Figure S1–2).

Truffle fruiting and tree growth season calculation

To calculate the lengths of the growing and fruiting seasons for host trees and truffles, S-shaped Gompertz functions were fit to the relationship between the numerical day of the year vs tree diameter and cumulative fruitbody production, respectively (Čufar et al., 2008). The Gompertz function is expressed as:

$$f'[t] + ae^{-be^{(-ct)}} \qquad \text{eq (1)}$$

Where *a*, *b*, and *c* are parameters that describe the maximum (*a*) and slope and timing (*b* and *c*) of tree growth or fruitbody production. These three parameters were fit for each site x year x tree combination (and site x year for truffle fruitbodies) using the least sum of squares method in R (nls function in the base package). The start, end, and point of max growth and fructification were calculated from these fit parameters using linear approximation of the Gompertz function. Accordingly,

Start day: $$-\frac{1}{c}\left\{1 + log\left[-\frac{1}{b}\right]\right\} \qquad \text{eq (2)}$$

End day: $$\frac{1}{c}\left\{e - 1 - log\left[-\frac{1}{b}\right]\right\} \qquad \text{eq (3)}$$

Max growth rate: $$\frac{ac}{e} \qquad \text{eq (4)}$$

Max growth day: $$\frac{1}{c}\left\{log\left[-\frac{1}{b}\right]\right\} \qquad \text{eq (5)}$$

For host trees, each individual tree was fit a different Gompertz function (for a maximum for 4 per site per year) and treated as a technical replicate. The average start and end days and max-growth-rates of all trees were computed as potential predictors of annual productivity.

Variance Partitioning

For each site x year combination, we summed the annual total number of fruitbodies per unit area (m^{-2} yr^{-1}). We then calculated mean truffle mass as the ratio of total annual mass (g yr^{-1}) over the total number of truffles (# of truffles yr^{-1}). This allowed us to express truffle yield (z, in g m^{-2} yr^{-1}) as the product of fruitbody yield (x, in # of fruitbodies m^{-2} yr^{-1}) and mean truffle mass (y, in g). In order to correct for the differences in scale between the variables x and y (measured in different units with multiplicative effects on z), we log-transformed the product z = x×y into the sum log(z) = log(x)+log(y). This allowed us to partition the variance in log(z) into the sum of two covariance terms (for x and y), such that

$$\mathrm{var}[\ \log(z)\ ] = \mathrm{cov}[\ \log(x), \log(z)\ ] + \mathrm{cov}[\ \log(y), \log(z)\ ].$$

When both covariance terms are positive (as they are in our analysis), then years with a greater truffle yield (z) tend to have both a greater number of truffles (x) and a greater mean truffle mass (y). This means that the ratio of the x and y covariance terms over var [log (z)] gives the proportion of the variance accounted for by x (truffle numbers) and y (mean truffle mass), respectively.

Variables and Model Selection

A large suite of predictors was assembled and exposed to model selection using the random forest algorithm. These were plotted in Figure 4a. We provide a full list of these variables along with the units and data source in Table S1.

Principal Component Analyses

For vegetative and soil meta-community variables principle component analyses (package "prcomp" in R) were performed to collapse the high dimensional datasets into three orthogonal axes (Supplemental Figures S1,S2).

For soil fungal meta-communities, there were 2,219 OTUs (after rarefaction and removal of singletons). This makes visualizing loading values with vectors on top of the PCA scatterplots difficult (particularly when points are annotated by site), due to the overlapping

labels. In Figure S2 (top row) we characterized the top 5 positive and negative loading values on the first 3 PCA axes.

For the dendrometer data, we conducted a separate principal component analysis. We included data for temperature and precipitation during the hottest quarter, mean tree growth during the hottest quarter, and mean tree water deficit during the warmest quarter (Figure 6a).

.

Supplemental Table 1. All variables used in the random forest analysis of annual truffle yield (g m^{-2} yr^{-1}). Variables are sorted according to the rank of their Inc Node Purity importance scores (as in the x-axis of Figure 3). The %MSE column gives another metric of variable importance (along with the numerical rank).

rank	variable	source	variable type	IncNod	%MSE (rank)
1	pH (in water)	in situ	soil	8424.14	12.11 (1)
2	bare soil (%)	in situ	ground cover	5334.86	10.79 (2)
3	precipitation sum of hottest quarter (mm)	ERA5	annual climate	3121.92	5.22 (4)
4	shrub cover (%)	in situ	vegetation	2405.20	7.43 (3)
5	mean temperature hottest quarter (°C)	ERA5	annual climate	1890.10	2.73 (14.5)
6	number of years harvested (yr)	instrumental	sampling	1827.13	0.21 (60)
7	herb cover (%)	in situ	vegetation	1688.63	4.71 (5)
8	soil CaCO3 (ppm)	in situ	soil	1306.72	4.45 (6)
9	samples (# of surveys yr^{-1})	Instrumental	sampling	993.73	-1.90 (63)
10	litter cover (%)	in situ	ground cover	661.81	3.94 (8)
11	precipitation sum of wettest month (mm)	WorldClim	climate means	354.73	1.89 (31)
12	total nitrogen (ppm)	in situ	soil	341.11	2.49 (18)
13	mean diurnal temperature range	WorldClim	climate means	332.22	2.73 (14.5)
14	total annual precipitation (mm)	WorldClim	climate means	320.94	2.72 (16)
15	coarse sand (ppm)	in situ	soil	311.33	2.86 (11.5)
16	precipitation sum of wettest quarter (mm)	WorldClim	climate means	293.82	3.13 (10)
17	exchangeable CaO (ppm)	in situ	soil	275.27	3.35 (9)
18	tree composition PC2	in situ	vegetation	274.44	2.59 (17)
19	organic carbon (ppm)	in situ	soil	254.13	2.32 (21)
20	mean min temperature of coldest month (°C)	WorldClim	climate means	222.87	4.05 (7)
21	shrub layer species richness (# of sp)	in situ	vegetation	221.96	2.86 (11)

22	fine sand (ppm)	in situ	soil	188.65	1.86 (32)
23	elevation (m a.s.l)	in situ	elevation	184.95	2.44 (19)
24	moss cover (%)	in situ	vegetation	176.34	2.34 (20)
25	mean temperature of wettest quarter (°C)	WorldClim	climate means	172.42	1.40 (51)
26	C:N ratio	in situ	soil	167.43	2.28 (22)
27	precipitation seasonality (c.o.v)	WorldClim	climate means	161.88	1.38 (52)
28	precipitation sum of driest quarter (mm)	WorldClim	climate means	157.85	1.44 (49)
29	soil fungi composition PC3	in situ	fungal community	140.07	2.07 (25)
30	organic matter (ppm)	in situ	soil	134.56	1.80 (35)
31	precipitation sum of warmest quarter (mm)	WorldClim	climate means	129.60	1.95 (28)
32	soil fungi composition PC2	in situ	fungal community	128.51	1.83 (33)
33	herb species richness (# of sp)	in situ	vegetation	128.29	1.79 (36)
34	soil P2O5 (Joret-Hebert extraction, ppm)	in situ	soil	125.21	1.56 (43)
35	plant species richness (# of sp)	in situ	vegetation	124.06	2.06 (26)
36	mean annual soil temperature (°C)	WorldClim	climate means	121.93	2.08 (24)
37	cation exchange capacity (meq)	in situ	soil	118.41	2.00 (27)
38	mean temperature of coldest quarter (°C)	WorldClim	climate means	115.96	1.93 (29.5)
39	exchangeable K2O (ppm).	in situ	soil	105.39	1.78 (37)
40	soil fungi composition PC1	in situ	fungal community	104.25	1.54 (44)
41	clay (ppm)	in situ	soil	103.08	1.93 (29.5)
42	isothermality	WorldClim	climate means	99.69	1.81 (34)
43	tree / shrub layer species richness (# of sp)	in situ	vegetation	93.93	0.36 (59)
44	temperature seasonality (std x 100)	WorldClim	climate means	92.02	2.21 (23)
45	precipitation sum of driest month (mm)	WorldClim	climate means	89.92	1.52 (46)
46	mean temperature of the hottest quarter	ERA5	climate means	89.44	1.35 (54)
47	tree composition PC1	in situ	vegetation	81.89	1.25 (56)
48	soil NO3-N (ppm)	in situ	soil	80.51	2.77 (13)

49	mean July soil temperature (°C)	WorldClim	climate means	75.54	1.76 (38)
50	precipitation sum of coldest quarter (mm)	WorldClim	climate means	70.06	1.41 (50)
51	tree species richness (# of sp)	in situ	vegetation	69.94	1.47 (48)
52	coarse silt (ppm)	in situ	soil	58.39	1.62 (41)
53	mean max temperature of hottest month (°C)	WorldClim	climate means	45.58	1.52 (46)
54	fine silt (ppm)	in situ	soil	44.84	1.68 (39)
55	tree composition PC3	in situ	vegetation	43.28	1.34 (55)
56	mean temperature of warmest quarter (°C)	WorldClim	climate means	42.29	1.14 (58)
57	NH4 - N on dry soil (ppm)	in situ	soil	32.02	-0.91 (62)
58	mean temperature of driest quarter (°C)	WorldClim	climate means	31.69	-0.06 (61)
59	soil exchangeable MgO (ppm)	in situ	soil	13.26	1.66 (40)
60	tree layer (%)	in situ	vegetation	12.25	1.15 (57)
61	mean annual temperature (°C)	WorldClim	climate means	11.34	1.61 (42)
62	mean annual temperature range (°C)	WorldClim	climate means	8.51	1.52 (46)
63	active soil CaCO3 (ppm)	in situ	soil	0.70	1.36 (53)

Supplemental Figures

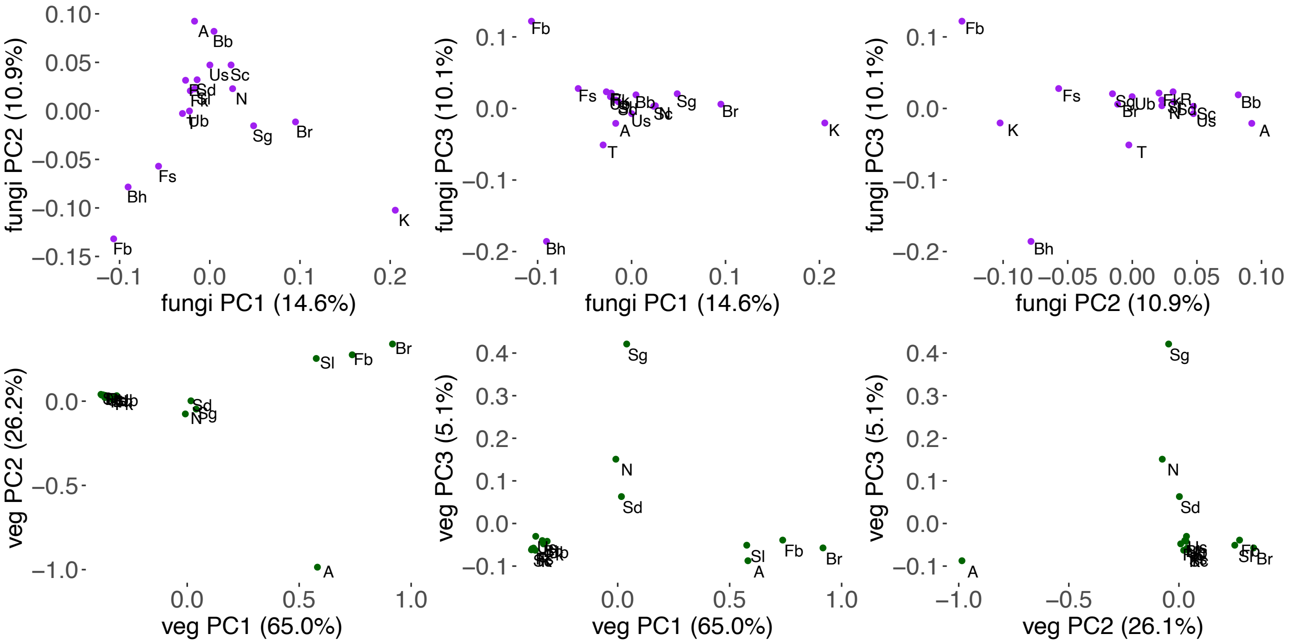

Figure S1. Pairwise scatterplots for all combinations of the first three principal components for soil fungal meta-community (top row) and tree species composition (bottom row). The % variability explained by each PC axis is listed parenthetically. Points are annotated by site name.

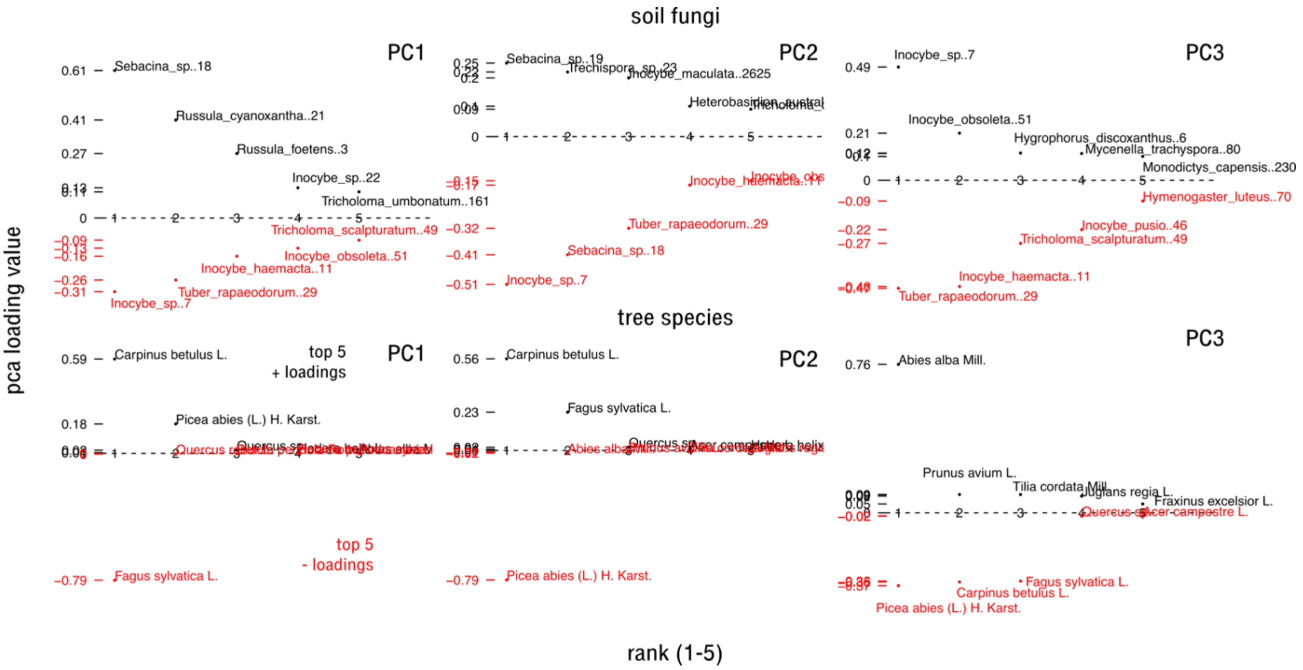

Figure S2. The top 5 positive and top 5 negative loading values for soil fungal meta-communities (top) and tree species composition (bottom) for the three PC axes.

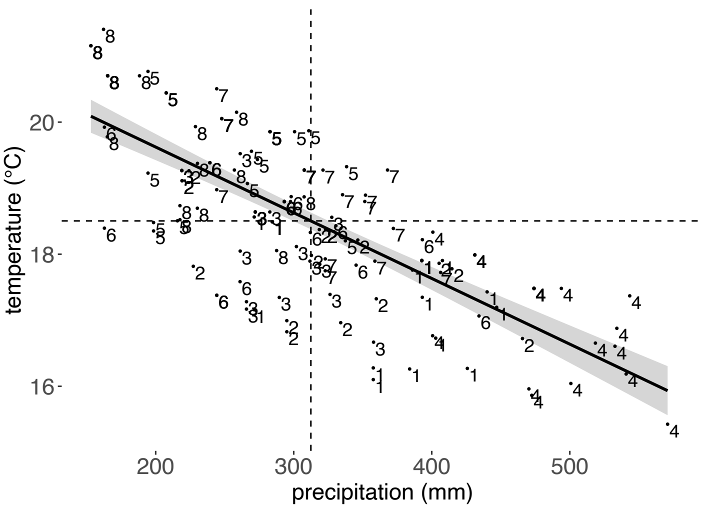

Figure S3. Temperature and precipitation during the hottest quarter are negatively correlated. Sites are annotated by year X (201X).

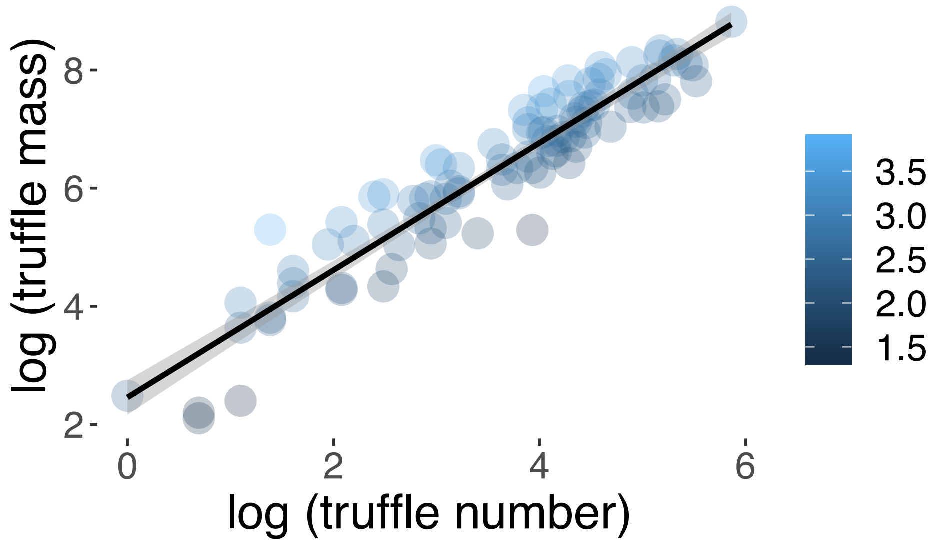

Figure S4. The log-transformed annual total number of truffles surveyed vs the total annual truffle mass with a linear best fit line ($p<0.001$, $R^2 = 0.89$). Points are colored according to the log-transformed mean mass of truffles.

Bibliography

Altschul, S. F., Gish, W., Miller, W., Myers, E. W., & Lipman, D. J. (1990). Basic local alignment search tool. *Journal of Molecular Biology*, *215*(3), 403–410. https://doi.org/10.1016/S0022-2836(05)80360-2

Čufar, K., Prislan, P., de Luis, M., & Gričar, J. (2008). Tree-ring variation, wood formation and phenology of beech (Fagus sylvatica) from a representative site in Slovenia, SE Central Europe. *Trees*, *22*(6), 749–758. https://doi.org/10.1007/s00468-008-0235-6

Edgar, R. C. (2013). UPARSE: Highly accurate OTU sequences from microbial amplicon reads. *Nature Methods*, *10*(10), 996–998. https://doi.org/10.1038/nmeth.2604

Moser, B., Büntgen, U., Molinier, V., Peter, M., Sproll, L., Stobbe, U., Tegel, W., & Egli, S. (2017). Ecological indicators of Tuber aestivum habitats in temperate European beech forests. *Fungal Ecology*, *29*, 59–66. https://doi.org/10.1016/j.funeco.2017.06.002
